# The inhibition of YTHDF3/m^6^A/LRP6 reprograms fatty acid metabolism and suppresses lymph node metastasis in cervical cancer

**DOI:** 10.7150/ijbs.87203

**Published:** 2024-01-12

**Authors:** Sheng Zhong, Quanwei Guo, Xiaona Chen, Xiaomin Luo, Yufei Long, Tuotuo Chong, Ming Ye, Hui He, Anwei Lu, Keyi Ao, Minuo Yin, Aimin Xu, Xin Li, Yi Hao, Xia Guo

**Affiliations:** 1 Shenzhen Key Laboratory of Viral Oncology; Department of Science and Innovation, Shenzhen Hospital, Southern Medical University, Shenzhen, China.; 2 The Third School of Clinical Medicine, Southern Medical University Guangzhou, China.; 3 Department of Thoracic Surgery, Shenzhen Hospital, Southern Medical University, Shenzhen, China.; 4 Department of Pathology, Affiliated Tumor Hospital of Xinjiang Medical University, Urumqi, China.; 5 Department of Pathology, Shenzhen Hospital, The University of Hong Kong, Shenzhen, China.; 6 Department of Obstetrics and Gynecology, Shenzhen Hospital of Southern Medical University, Shenzhen, China.; 7 Department of Medicine, University of Hongkong, Hongkong, China.; 8 Department of Ultrasound, South China Hospital of Shenzhen University, Shenzhen, China.

**Keywords:** cervical cancer, YTHDF3, m^6^A, lymph node metastasis, LRP6, fatty acid metabolism

## Abstract

The lipid synthesis of fatty acid (FA) represents a significant hallmark in the occurrence and progression of malignant tumor, which are associated with lymph node (LN) metastasis. Elucidation of the molecular mechanisms underlying LN metastasis could provide therapeutic strategies for cervical cancer (CCa). N6-Methyladenosine (m^6^A), the most prevalent and abundant RNA modification, exerts specific regulatory control over a series of oncogene expressions. This study demonstrated a clinical correlation between the upregulation of the m^6^A reader YTHDF3 and LN metastasis, thereby contributing to poor overall survival probability (OS) among CCa patients. The mechanistic investigation revealed that SREBF1 transcriptionally activated YTHDF3 expression by binding to its promoter. Functional experiments demonstrated that the upregulation of YTHDF3 significantly enhanced the *in vitro* proliferative, migratory, and invasive capacities of CCa cells, while also promoting lymphangiogenesis and facilitating LN metastasis *in vivo*. Mechanistically, the upregulation of LRP6 through YTHDF3-mediated m^6^A modification resulted in increased expression of FASN and ACC1, leading to both lipolysis of lipid droplets and synthesis of free fatty acid. Ultimately, this promoted fatty acid metabolism and enhanced LN metastasis by activating the LRP6-YAP-VEGF-C axis, which could induce lymphangiogenesis in CCa. Our study highlighted that YTHDF3 can serve as a promising therapeutic target and predictive biomarker for CCa patients with LN metastasis.

## Introduction

Cervical cancer (CCa) is ranked as the fourth most prevalent malignant tumor, posing a formidable threat to women's lives worldwide 1. LN metastasis is a crucial factor that significantly impacts recurrence and overall survival in CCa patients with the 5-year overall survival rate dropping from 95% to 51% 2,3. Therefore, it is imperative to elucidate the mechanism of LN metastasis for potential therapeutic interventions in CCa.

The N6-Methyladenosine (m^6^A) modification has emerged as the most frequent RNA modification in eukaryotic mRNAs and exerts regulatory control over gene expression at the post-transcriptional level 4,5. This modification is responsible for the stability, splicing, and translation of modified mRNAs, which are essential for a variety of physiological and pathological. processes. The YT521-B homology (YTH) domain proteins (YTHDF1, YTHDF2, YTHDF3, YTHDC1) serve as m^6^A "readers" 6. Among them, YTHDF3 functions as a pivotal factor for fine-tuning between YTHDF1 and YTHDF2 7, revealing its oncogenic function in the progression of various cancers 8-11.

Reprogramming cellular energy metabolism is increasingly acknowledged as a hallmark of cancer 12. Cancer cells must overcome nutrient and energy deficits, ultimately surviving metastasis by rewiring metabolic programs 13. Recent studies demonstrated that the amplification of lipid synthesis, particularly fatty acid (FA) production, plays a crucial role in the development and progression of malignant tumors, especially those involving LN metastasis 14. Recent studies proposed the concept that free FA metabolism fostered LN metastasis in CCa 15 by facilitating the formation of pre-metastatic niches within lymph nodes and inducing lymphangiogenesis 16. Nevertheless, the precise mechanism underlying how CCa remodels lipid metabolism to facilitate this process remains incompletely understood.

Here, we conducted comprehensive analyses using high-throughput sequencing and phenotype experiments to investigate the role and epigenetic mechanisms of m^6^A reader YTHDF3 in CCa progression. Our findings suggested that YTHDF3 was a potential independent predictor contributing to LN metastasis in CCa. Moreover, we interrogated the mechanism by which YTHDF3 facilitated LRP6 translation efficiency by binding to the m^6^A modification site on LRP6 mRNA. Elevated levels of YTHDF3 promoted lipolysis and fatty acid (FA) synthesis, leading to the induction of lymph node (LN) metastasis through the activation of the LRP6-YAP-VEGF-C axis. Our research uncovered significant prognostic and therapeutic implications of YTHDF3 in CCa, highlighting the potential for suppression of LN metastasis by inhibiting YTHDF3/m^6^A/LRP6.

## Methods

### Tissue microarray (TMA)

In this study, the TMA included 144 clinical samples (17 cervical intraepithelial neoplasia, 95 cervical squamous cancer, and 32 normal cervical epithelial epithelium). Shanghai OUTDOB Biotech Co was responsible for constructing the TMA and collecting the associated patient information (Shanghai, China) in** Table 1 and Table 2.**

### Cell Lines and Cell Culture

The HFF-1 (Human epithelial foreskin fibroblast cells), SiHa, and CaSki cell lines were obtained from the Cell Bank of the Chinese Academy of Sciences (Shanghai, China). Human lymphatic endothelial cells (HLECs) were maintained by endothelial cell media from ScienCell (CA) and were obtained from iCell Bioscience Inc (Shanghai, China). The SiHa and CaSki cells were incubated in 1640 medium (Gibco, USA), meanwhile the HFF-1 cell line was grown in DMEM medium (Gibco, USA). All of the medium was augmented with 10% fetal bovine serum (Excell, Uruguay), 100 U/mL penicillin, and 100 U/mL streptomycin (BI, USA). Cells were incubated in a humidified incubator at 37°C with 5% CO_2._ The short tandem repeat (STR) genotyping was confirmed for these cell lines. Furthermore, Haixing Biosciences (Guangzhou, China) used CRISPR-Cas9 technology to construct knockout YTHDF3 SiHa cells (YTHDF3^-/-^ SiHa). The specific sgRNAs were listed in the **Supplementary Table 2**.

### Immunohistochemistry (IHC) staining and scoring analyses

The clinical samples were analyzed with the kit, PV-6000 (Zhongshan Jinqiao, Beijing) for the instructions. Paraffin-embedded tissues were dewaxed and the antigens were repaired to 120°C for 10 min. To prevent endogenous peroxide activity, the samples were kept in the solution and then washed with PBS buffer. Appropriate types of primary antibodies were used in the corresponding pH unmasking solutions. The tissues were then exposed to a primary antibody at 4°C for one night. Next, the corresponding secondary antibodies were utilized in the samples at 37°C for 1 h after washing with PBS buffer. Then, it was separately stained for hematoxylin (SP9001, ZSZB-Bio, Beijing, China) and 3, 3-diaminobenzidine (DAB) (Zhongshan Jinqiao, Beijing) for 2-5 min. After the tissues were immersed in water for 20 min, we used a Leica DMI8 microscope to record all tissue sections. Two senior pathologists independently inspected and graded IHC stained sections and the signalling intensity and staining distribution ratings were multiplied to arrive at a final score. The distribution values were calculated by considering the percentage of positive cells as follows: 1 (5%—25%), 2 (25%—50%), and 3 (50%—75%). The cut-off value was defined as the median. The **Supplementary Table 1** provided specifics on the primary antibodies.

### Cell transfection

The density of 2*10^5^ cells was transfected with plasmids by Lipofectamine 3000 reagent (Invitrogen, USA). Samples were collected for RNA or protein extraction after 36-48 h transfection, respectively.

### Lentivirus vector and cell infection

Short hairpin RNAs (shLRP6, shYTHDF3, shNC) and overexpressed YTHDF3 lentivirus were bought from Gene Chem (Shanghai, China) and stably transfected into CaSki and SiHa cells. The transfected cell lines were added to the Hitrans G and the Puromycin (Beytime, 2 μg/mL) was administered for a week. Finally, the stable knockdown of YTHDF3 or LRP6 cell lines was established successfully.

### RNA extraction and quantitative RT-PCR analysis

Trizol (Invitrogen, USA) was employed to retrieve RNA from cells and tissues. For cDNA synthesis, RNA (1 μg) were reverse transcripted using a Reverse Transcription Kit (YEASEN, Shanghai). The PCR amplification parameters consisted of an initial denaturation at 95°C for 32 s, followed by an additional denaturation step at 95°C for 2 min, and finally an annealing step at 60°C for 32 s for a total of 40 cycles. The amplification was performed on the 7500 RT-PCR instruments (Applied Biosystems, USA) using the SYBR Green Master Mix (YEASEN, Shanghai). All experiments were done independently three times and the primers are displayed in the **Supplementary Table 2**.

### Immunofluorescence (IF)

Cells were harvested when they achieved 70% confluency in six-well plates. Samples were treated with 4% paraformaldehyde and permeabilized using 0.5% TritonX-100. Following this, the blocked samples using 10% goat serum. The YTHDF3 antibody was then added and allowed to incubate with the samples at 4°C overnight. On a subsequent day, the samples underwent incubation with Alexa Fluor 647 (Goat Anti-Rabbit immunoglobulin G), and the nuclei were stained with DAPI while imaging the fluorescence microscope.

### Protein extraction and western blotting

Protein was extracted from cells using RIPA buffer (Beyotime, China) and added with protease inhibitor or phosphatase inhibitor (Applied Biosystems, USA). The protein concentration was determined by the Bicinchoninic Acid Protein Assay Kit (EpiZyme, China) and was diluted in 5 × loading buffer (Legend, China) and boiled for 10 min at 95°C Following a one-hour blocking step at room temperature using 5% milk, the primary antibodies were incubated overnight at 4°C. Subsequently, the 1 × TBST buffer was used to wash the samples, which were later incubated with the corresponding secondary antibodies for one hour at 37°C. The Chemidoc Touch (Bio-Rad, USA) visualized the blots via an ECL reagent (Bio-Rad, USA). Additional information on the primary antibodies is listed in **Supplementary Table 1**.

### Cell proliferation assay

The intensity of 3*10^3^ cells was seeded on 96-well plates and sustained in the cell culture incubator with 5% CO_2_. The OD value was subsequently examined by the Cell Counting Kit 8 (Meilun, China) at different times, including 0 h, 24 h, 48 h, and 96 h. A microplate reader (Bio-Tek) set to 450 nm was used to gauge each well's absorbency.

### Colony formation assay

In 6-well plates, 3*10^3^ cells were seeded for 2 weeks. Following fixation with 4% formaldehyde for 30 min, colonies were treated with 0.5% crystal violet for 20 min before being washed with PBS.

### Quantification of the number of lipid droplets

Cells were plated on 6-well plates with sterile glass coverslips and fixed in PBS containing 1 ml of 4% formaldehyde for 10 min once they reached 60%-80% confluence. Nile Red dye (Solarbio, China) with the cells were co-incubated for 10 min and was imaged using a 561 nm laser for excitation and a 617 nm emission filter. Frozen tissues and cells were rapidly fixed with 4% formaldehyde and washed with 60% isopropanol before being stained for 15 min with a freshly prepared working solution of Oil Red O. Optical microscopy was used to observe the number of lipid droplets.

### Cell migration and invasion assay

A total of 4*10^5^ cells were distributed onto 6-well plates until they reached a confluence of 95%. Then, an artificial scratch-presenting wound was constructed in the cell plate and imaged on a Leica DMI8 microscope. Twenty-four hours later, the initial injuries were once again captured, and Image J was used to examine the surface area of the wound. In the invasion experiment, 5*10^4^ cells were placed in each well of a 24-well plate with 8 μm pore size. Cells with a medium free of serum were placed in the top compartment meanwhile the 15% FBS media was put in the bottom chamber. The chamber was taken out after the membrane had been incubated for 14 h and gently washed its remnant by PBS. Each chamber was separately fixed with paraformaldehyde and dyed with 0.25% crystal violet. Samples were recorded on a Leica DMI8 microscope, and Image J was used for analysis.

### mRNA stability assay

Cells were plated on 6-well plates until approximately 50% confluent before being treated with Actinomycin D (4 µg/mL) (Sigma, USA) at 0, 6, and 9 h. Total RNA was extracted and performed by qRT-PCR. These mRNA values for each group were normalized by GAPDH.

### Nascent protein synthesis assays

SiHa cells were incubated in L-homopropargylglycine (HPG) at 37°C for 30 min. After being fixed with the 3.7% formaldehyde, the cells were washed twice with 3% BSA and followed by 0.5% Triton X-100 for 20 min. The samples were eventually incubated with the cocktail mix for 30 min and recorded by the Lecia microscope.

### HLECs tube formation assay

The YTHDF3^-/-^ group and control group were purified by using ultra-filtration spin columns from Millipore in the United States. The HLECs were seeded and co-cultured with the concentrated supernatant for 8 h on the 96-well plates that were pre-coated with Matrigel (Corning, USA). A Leica DMI8 microscope recorded tubes and the intensity of length was measured by Image J.

### Xenograft and LN metastasis models

Female BALB/c nude mice (4-6 weeks old, 18-20 g) were housed in an SPF environment in the Southern Medical University Animal Center. These nude mice were divided into different groups at random (n = 6). The subcutaneous nude mice received injections of differential cells (1*10^7^ cells per mouse) (SiHa/OE-YTHDF3 SiHa/YTHDF3^-/-^SiHa). The volume of the tumor was measured every three days through the formula lengthwidth^1/2^ and the luciferase signal intensity was recorded every week. Then, on the twenty-first day following vaccination, each group was sacrificed. For the LN metastasis model, cells (1*10^7^ cells per mouse) were interposed directly to the mouse footpad (n = 5). Every group were sacrificed on the 30^th^ day following the vaccination. IHC analysis and H&E staining were performed on each dissected tumor and lymph node.

### ATAC-sequencing

For the transposes-accessible chromatin with high-throughput sequencing (ATAC-seq), Being treated with the lysis buffer, the cells were washed using PBS. Subsequently, a Nextera enzyme (Illuminsa, San Diego, CA) was employed for labelling, followed by PCR amplification consisting of 10-12 cycles using primers. Paired-end sequencing (Illuminsa, San Diego, CA) was performed with 2*50 cycles. To ensure comprehensive analysis, we integrated overlapping regions observed across all replicates while disregarding Chrome regions and those that overlapped ENCODE blacklisted regions. All sequencing was carried out with two biological replicates.

### CUT&Tag

The total DNA was fragmented and immunoprecipitated using a (Millipore, Bedford, MA). qRT-PCR quantified these fragments after being immunoprecipitated by the anti-SREBF1 and anti-IgG antibodies. Details of the primary antibodies employed are given in **Supplementary Table 1**. The sequences of the primers are listed in the **Supplementary Table 2.**

### Dual-luciferase reporter assays

Dual-luciferase reporter assays were performed on HEK 293T cells. For the full-length SREBF1 plasmids, the coding sequence was cloned using the pcDNA3.1 vector. YTHDF3 promoter region was cloned into pGL3-basic (Ruibo, China) before being constructed the plasmid. Additionally, we established the mutant YTHDF3 promoter plasmid according to the ATAC-seq data. HEK-293T cells were plated in 24 wells (6*10^4^ cells per well) and transfected with these plasmids using Lipofectamine 3000 regent (Invitrogen, USA). After transfected 48 h, Fluc and Rluc activities were measured by a Dual-Luciferase Reporter Assay System (YESEN, Shanghai) according to the manufacturer's instructions.

### RNA-sequencing

The samples consisted of SiHa, HFF-1, and YTHDF3^-/-^ SiHa cells. Total RNAs of cells were extracted by the Trizol Reagent and the mRNA sequencing was simultaneously performed the Kangcheng (Guangdong, China). Reads were aligned to the human ensemble genome GRCh38 using the Hisat2 aligner (ver. 2.1.0) under appropriate parameters.

### MeRIP sequencing and MeRIP quantitative PCR

TRIzol (Invitrogen, USA) was extracted from the total RNA. The MeRIP sequencing was responsible for the Cloud See Biotech (Guangzhou, China). First, the mRNA was broken up for a precise procedure, and then it was exposed to an m^6^A antibody (Abcam, USA) for immunoprecipitation. Using feature Counts (ver. 1.6.3), the reads that mapped the genome. The R software was used for differential gene expression analysis with an FDR of 0.05. For MeRIP-qPCR experiments, the total RNA (300 μg) was extracted, fragmented and immunoprecipitated by Magna-MeRIP m^6^A kit (Millipore, USA) and the input was normalized to the result. **Supplementary Table 2** contains a list of the primers for qPCR analysis.

### RNA immunoprecipitation and high-throughput sequencing

The samples were collected by centrifugation at 12,000 g for 10 min after being harvested using the icy IP lysis buffer. It was mixed with 30 μL protein G beads (Invitrogen, USA). The mixture was co-incubated with YTHDF3 antibodies overnight at 4°C. Subsequently, the samples were extracted co-precipitated RNAs by Trizol and ethanol-precipitated with glycogen to obtain the products. The final products were used for the RIP-qPCR and were sequenced by Gzscbio (Guangzhou, China).

### Statistical analysis

With the Department of Bioinformatics' guidance, statistical analyses were carried out. Each image includes information on the sample size (n), statistical test run, and accompanying P-values. The primary findings were examined with GraphPad Prism 8.0 (GraphPad Software, Inc) and the results were presented as the mean SEM or mean SD. The evaluation of differences primarily relied on the implementation of either the two-tailed t-test for students or the one-way ANOVA analysis. Additionally, the correlation between the expression of YTHDF3 proteins in histological subtype and the LN metastasis status of patients was analyzed by the Pearson Chi-square test. *P < 0.05, *P < 0.01, ***P < 0.001, ****P < 0.0001, ns: not significant.

## Results

### YTHDF3 is upregulated in CCa and essential for CCa progression and LN metastasis

**Figure 1:**
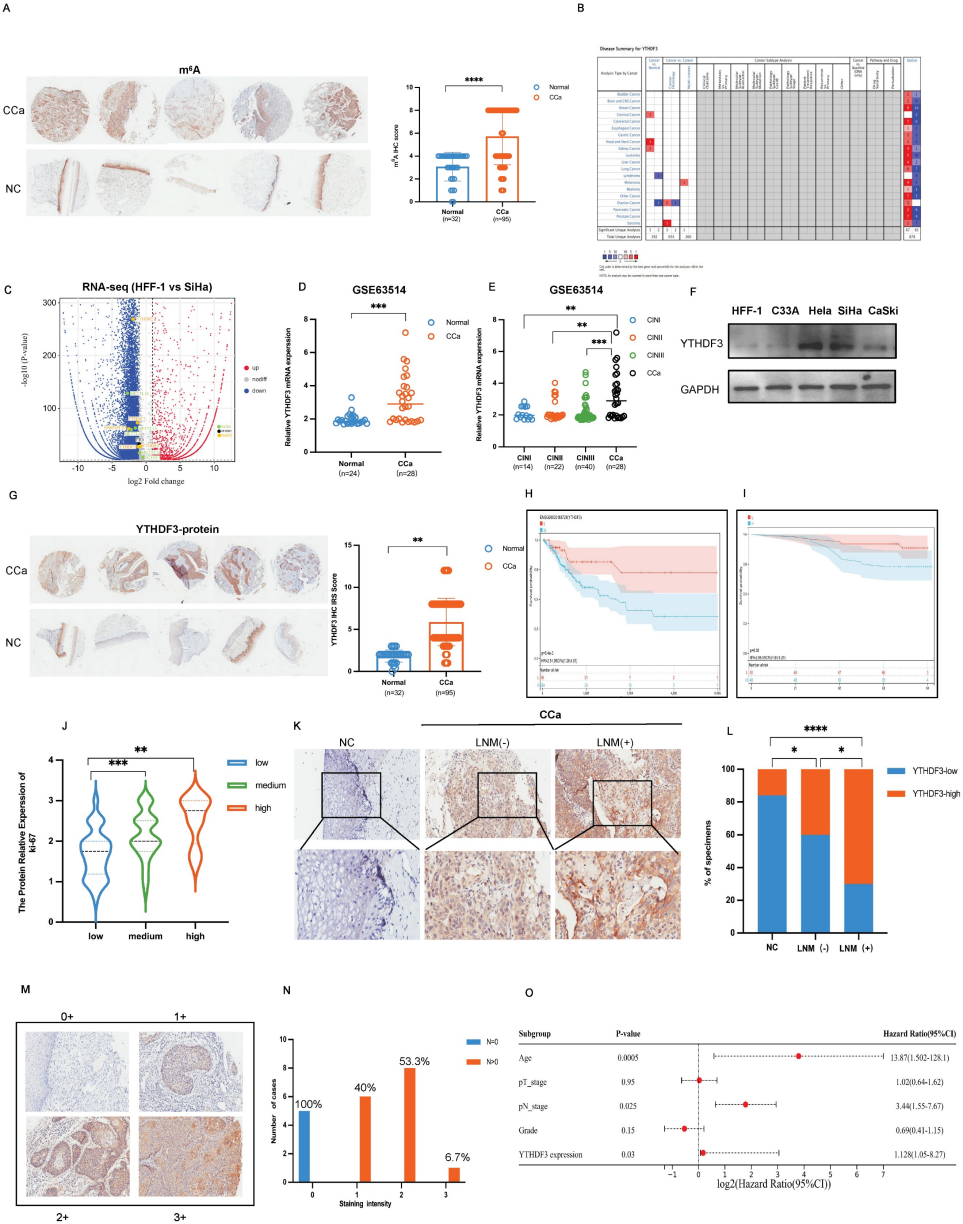
** Expression and clinical significance of m^6^A reader YTHDF3 and its correlation with LN metastasis of CCa. (A**) Representative IHC images on the tissue microarray (TMA) showed the m^6^A expression in the CCa and normal cervical tissue (NC). The tissue microarray (TMA) included 17 cervical intraepithelial neoplasia samples, 95 cervical cancer samples and 32 normal control samples. **(B)** The Oncomine analyzed that YTHDF3 is highly expressed in the CCa. **(C)** The volcano map showed the relative RNA expression of the m^6^A regulators in the comparison of SiHa and HFF-1 cells and the expression of YTHDF3 was upregulated in SiHa cells (reader: YTHDC2, YTHDC1, YTHDF3, YTHDF2, HNRNPCP2 (orange); writer: METTL16, METTL13, METTL4, METTL14, ZC3H13 (green); eraser: FTO (black)).** (D)** YTHDF3 mRNA was highly expressed in CCa (n = 28) compared with NC (n = 24) in the GEO datasets (GSE63514) by the t-test. **(E)** The degree of cervical intraepithelial neoplasia (CIN) was gradually associated with the expression of YTHDF3 in the GSE63514 by the one-way ANOVA. **(F)** Western blotting showed an increase in YTHDF3 expression in CCa cell lines (SiHa, CaSki, Hela) compared with the noncancerous control (HFF-1). **(G)** Representative IHC images on the tissue microarray (TMA) showed higher YTHDF3 expression in the CCa (5 cases) compared with normal cervical tissue (NC) (5 cases). The tissue microarray (TMA) included 17 cervical intraepithelial neoplasia samples, 95 cervical cancer samples and 32 normal control samples. **(H)** Kaplan-Meier survival analysis through Sanger Box displayed the YTHDF3 expression (low, n = 69; high, n = 204) and overall survival rate of CCa patients in the TCGA dataset by the log-rank test; *P* = 0.0054. **(I)** Kaplan-Meier survival analysis through Sanger Box showed the YTHDF3 expression (low, n = 50; high, n = 48) and the overall survival rate of CCa patients in the TMA by the log-rank test; *P* = 0.03. **(J)** The one-way ANOVA analysis demonstrated that there was a positive correlation between the expression of YTHDF3 and ki-67. **(K-L)** Representative images of IHC and percentages indicated the YTHDF3 expression in the CCa tissues with or without LN metastasis and normal cervix. Chi-square tests were used in this study. **(M-N)** IHC analysis of YTHDF3 on CCa samples performed the score of YTHDF3 staining. Scoring of YTHDF3 staining intensity in 5 CCa without LN metastasis and 15 tumour specimens with LN metastasis (0, no; 1: weak; 2: moderate; 3: strong expression). **(O)** Multivariate Cox regression analysis of clinical parameters identified the independent prognostic factors of CCa which are YTHDF3 expression, LN metastasis, and age. The data are shown as the means ± SDs; *P < 0.05; **P < 0.01; *** *P <* 0.001; **** *P <* 0.0001.

Recent investigations revealed the indispensable role of m^6^A modification in tumorigenesis, particularly in regulating metastasis in various cancers 17,18. However, the precise function of m^6^A modification in the LN metastasis with CCa remains elusive. Firstly, we utilized the tissue microarray (TMA) and immunohistochemistry (IHC) assays to evaluate the m^6^A levels in samples comprising 17 cervical intraepithelial neoplasia samples, 95 cervical cancer samples, as well as 32 matched adjacent-tumour controls. Notably, the level of m^6^A methylation was dramatically increased in CCa tissues (*P* < 0.0001; Fig. 1A). The Oncomine database illustrated a remarkable upregulation of YTHDF3 expression in CCa (Fig. 1B). Moreover, RNA-seq analysis of the SiHa and HFF-1 cells showed the relative mRNA expression of m^6^A regulators (YTHDC2, YTHDC1, YTHDF3, YTHDF2, HNRNPCP2, FTO, METTL3, METTL14, ZC3H13, METTL16, METTL14) and showed that YTHDF3 expression was significantly increased in SiHa cell compared to HFF-1cell (Fig. 1C).

To further validate the correlation between YTHDF3 upregulation and CCa, we scrutinized the GSE63514 dataset and ascertained that YTHDF3 mRNA expression was remarkably elevated in CCa tissues compared to the normal cervical epithelium (n = 24, *P* < 0.001) (Fig. 1D).

Additionally, there was a positive correlation between increased YTHDF3 levels and the degree of cervical intraepithelial neoplasia (CIN) (*P* < 0.001, Fig. 1E). Western blots confirmed that the upregulation of YTHDF3 was exhibited in CCa cell lines (SiHa, CaSki, Hela) compared with the normal control cell line (HFF-1) (Fig. 1F). TMA results showed a conspicuous overexpression of YTHDF3 in CCa tissues when compared to normal cervical tissues (Fig. 1G). Furthermore, Kaplan-Meier survival analysis of the Cancer Genome Atlas (TCGA) database demonstrated that an increased expression of YTHDF3 significantly reduced overall survival (OS) times (*P* = 0.0054, Fig. 1H). Similarly, the upregulation of YTHDF3 led to a poor prognosis in CCa patients in TMA analysis *(*Fig. 1I, Fig. S1A) (n = 92,* P <* 0.05). Our TMA analysis further revealed a positive correlation between YTHDF3 and ki-67 expression, as well as m^6^A modification level (Fig. 1J, Fig. S1B). These findings collectively indicated that YTHDF3 was upregulated in human CCa cells and tissues alongside its corresponding m^6^A methylation level.

To further explore the potential roles of YTHDF3 in LN metastasis, we employed immunohistochemistry (IHC) assays to assess YTHDF3 expression in CCa tissues with and without LN metastasis. We observed a slight increase in YTHDF3 expression in CCa tissues without LN metastasis (LNM^-^), whereas those from patients with LN metastasis (LNM^+^) displayed a remarkable upregulation of YTHDF3 protein level (Fig.1K-L). Furthermore, the higher staining intensity of YTHDF3 was evaluated in 20 LNM^+^ specimens, all classified as T2 and T3 clinical stages (Fig. 1M-N). Based on these findings, we concluded that YTHDF3 expression gradually increased in CCa patients with LN metastasis. Subsequently, a multivariate Cox regression analysis incorporating complete clinicopathological characteristics identified age (*P* = 0.0005), pathological N stage (*P* = 0.025), and YTHDF3 expression (*P* = 0.03) as significant independent prognostic factors for CCa patients' outcomes (Fig. 1O). Taken together, these findings implied that the upregulation of YTHDF3 played a crucial role in the malignant process and LN metastasis of CCa.

### Transcription factor SREBF1 activates *YTHDF3* transcription in CCa

**Figure 2:**
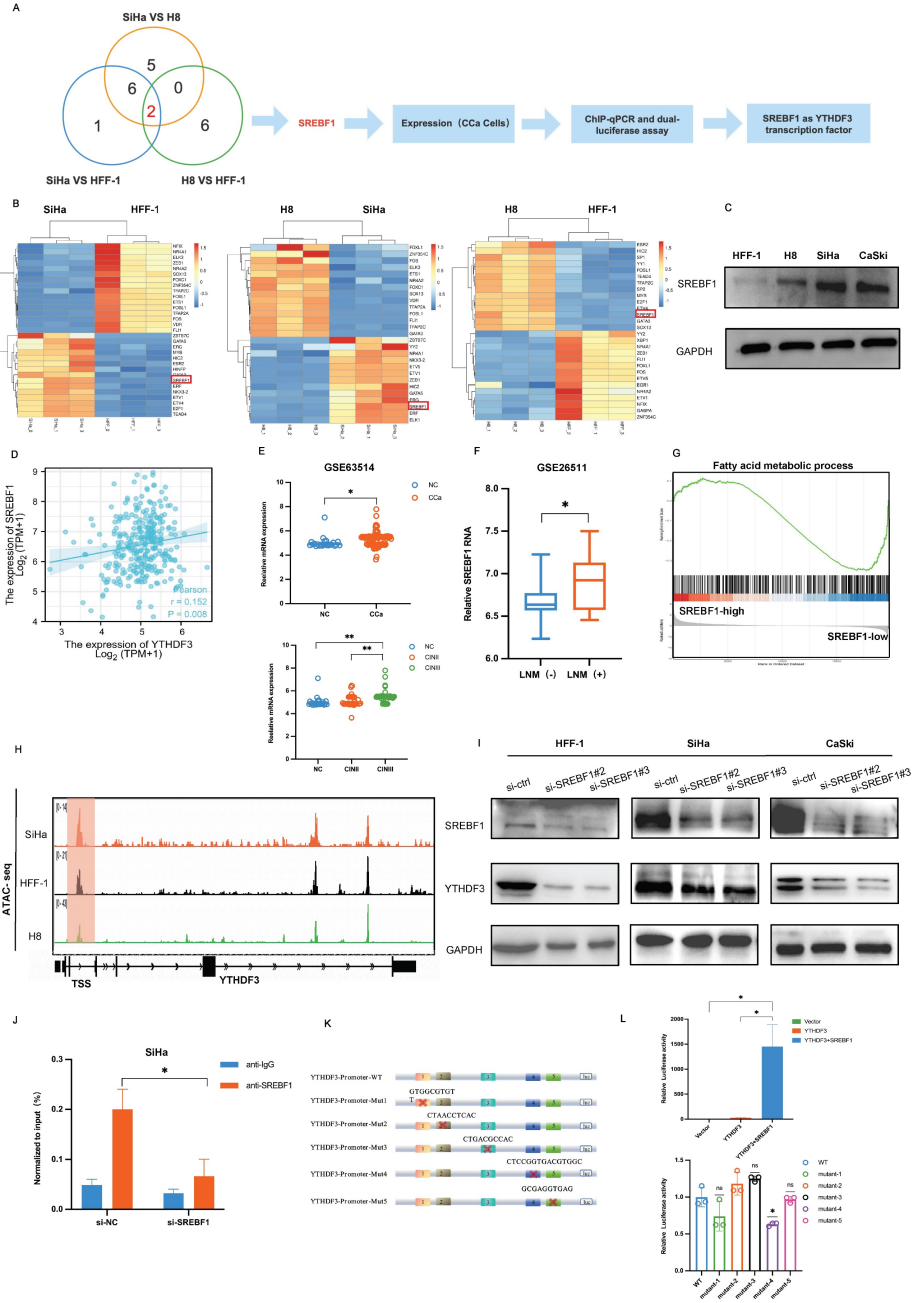
** SREBF1 is a transcription factor that activates YTHDF3 transcription in CCa. (A)** A graphic illustration performed the process of selection SREBF1 which as a transcript factor activated YTHDF3 transcription in CCa.** (B)** Heatmap of RNA-seq showed that differentially expressed transcript factors may regulate YTHDF3 transcription in different comparisons (SiHa vs. HFF-1, SiHa vs. H8 and H8 vs. HFF-1).** (C)** Western blots showed the expression of SREBF1 in the CCa cell (SiHa, CaSki), noncancerous cell line with HPV16 E6 and E7 (H8), and noncancerous control (HFF-1). **(D)** Pearson correlation confirmed the positive association between YTDHF3 and SREBF1 in CCa through the Xiantao website. **(E)** The SREBF1 mRNA was highly expressed in CCa (n = 28) compared with NC (n = 24) in the GEO datasets (GSE63514).** (F)** GSE26511 showed a higher expression of SREBF1 in CCa with LN metastasis^+^ than in LN metastasis^-^. **(G)** GSEA analysis showed the DEGs were enriched in the Fatty acid metabolic process in the SREBF1^ high^ group. **(H)** IGV plots of ATAC-seq showed that the transcription starts site signal of YTHDF3 in the SiHa, H8 and HFF-1 cells. the orange distribution represented SiHa cells, the black distribution represented HFF-1 cells, and the green distribution represented H8 cells.** (I)** Western blots validated the knockdown efficiency of SREBF1 and the expression of YTHDF3 after being transfected with si-SREBF1 in the SiHa, CaSki, and HFF-1 cells, **(J)** CUT&Tag assays verified that the enrichment of YTHDF3 was underregulated in the si-SREBF1 group.** (K-L)** Dual luciferase assay showed the relative luciferase activities in the different groups (Vector, YTHDF3, YTHDF3+SREBF1, YTHDF3 mutants+SREBF1). The ratios of Renillaluciferase were normalised to the control mimics for each group. The figure is depicted in Figdraw. The data are shown as the means ± SDs; *P < 0.05; **P < 0.01; *** *P <* 0.001; **** *P <* 0.0001.

The ATAC-seq has emerged as an invaluable, versatile, and widely adaptable method for profiling accessible chromatin regions, enabling the identification of variable transcription factor (TF) motif accessibility in eukaryotes 19. To clarify the mechanism underlying the YTHDF3 upregulation in CCa, we employed a combination of RNA-seq and ATAC-seq analysis in three cell lines: HFF-1 (Human epithelial foreskin fibroblasts cell line), H8 (immortal cervical epithelium cell line), and SiHa (cervical cancer cell line). Our sequencing results demonstrated that SREBF1 exhibited the most pronounced differential expression among these cells (Fig. 2A). Moreover, The RNA-seq heatmap showed a significant upregulation of SREBF1 in SiHa compared to control cell lines (HFF-1 and H8) (Fig. 2B). Consistent results were observed through western blot analysis in HFF-1, H8, SiHa and CaSki cell lines (Fig. 2C). Additionally, Pearson correlation analysis of TCGA datasets unveiled a positive correlation between YTHDF3 and SREBF1 expression levels (Fig. 2D).

To assess the impact of SREBF1 on CCa progression, we observed an upregulation of SREBF1 in CCa tissues compared to normal cervical cancer using the TCGA database and GEO database (Fig. 2E, Fig. S1C). Furthermore, the Kyoto Encyclopedia of Genes and Genomes (KEGG) analysis demonstrated that the DEGs in the SREBF1^high^ and SREBF1^low^ groups were enriched in the PI3K and MAPK pathways Fig. S1D). GSEA analysis of GSE26511 revealed that the RNA level of SREBF1 was significantly elevated in CCa tissues with LN metastasis compared to those without LN metastasis (*P <* 0.05; Fig. 2F). GSEA analysis of GSE63514 highlighted a significant enrichment of SREBF1-associated genes involved in the Fatty acid (FA) metabolic process (Fig. 2G), suggesting a positive correlation between SREBF1 and FA metabolism. Additionally, we investigated the chromatin accessibility of YTHDF3 at transcription start sites using ATAC-seq in HFF-1, H8 and SiHa cell lines. The IGV image demonstrated that CCa cells exhibited significant alterations in chromatin accessibility (Fig. 2H).

To confirm SREBF1 as a TF regulating YTHDF3 expression, two distinct small interference sequences targeting different sites (si-SREBF1#2, si-SREBF1#3) were transfected into HFF-1, SiHa and CaSki cell lines. Western blots results indicated that SREBF1 deficiency led to a reduction in the expression of YTHDF3 (Fig. 2I). Furthermore, we performed CUT &Tag assays and found that the SREBF1 silence could effectively decrease the enrichment of YTHDF3 in CCa cell (Fig. 2J). Importantly, we constructed the YTHDF3 promoter-luciferase plasmid, pRL-TK (renilla luciferase plasmid), an SREBF1 overexpression plasmid or empty vector (pcDNA3.1) plasmid. The dual luciferase activity assay demonstrated a remarkable surge in luciferase activity upon co-transfection with the YTHDF3 promoter plasmid and SREBF1 overexpression plasmid when compared with the vehicle control group and only the YTHDF3 promoter group (Fig. 2K-L). Furthermore, the binding sites were predicted based on sequencing results and YTHDF3 mutation plasmids were constructed, which were transfected with SREBF1 plasmids into the cell separately. The dual luciferase activity assay indicated the luciferase signal was downregulated in the mutant-4 group compared with the WT- group (Fig. 2L), which identified the site of SREBF1 binding to YTHDF3. In summary, we substantiated that SREBF1 functioned as a TF binding to the promoter of YTHDF3 and further stimulated its transcriptional activation in CCa.

### YTHDF3 promotes CCa cell proliferation, invasion, and HLECs lymphangiogenesis *in vitro*

**Figure 3:**
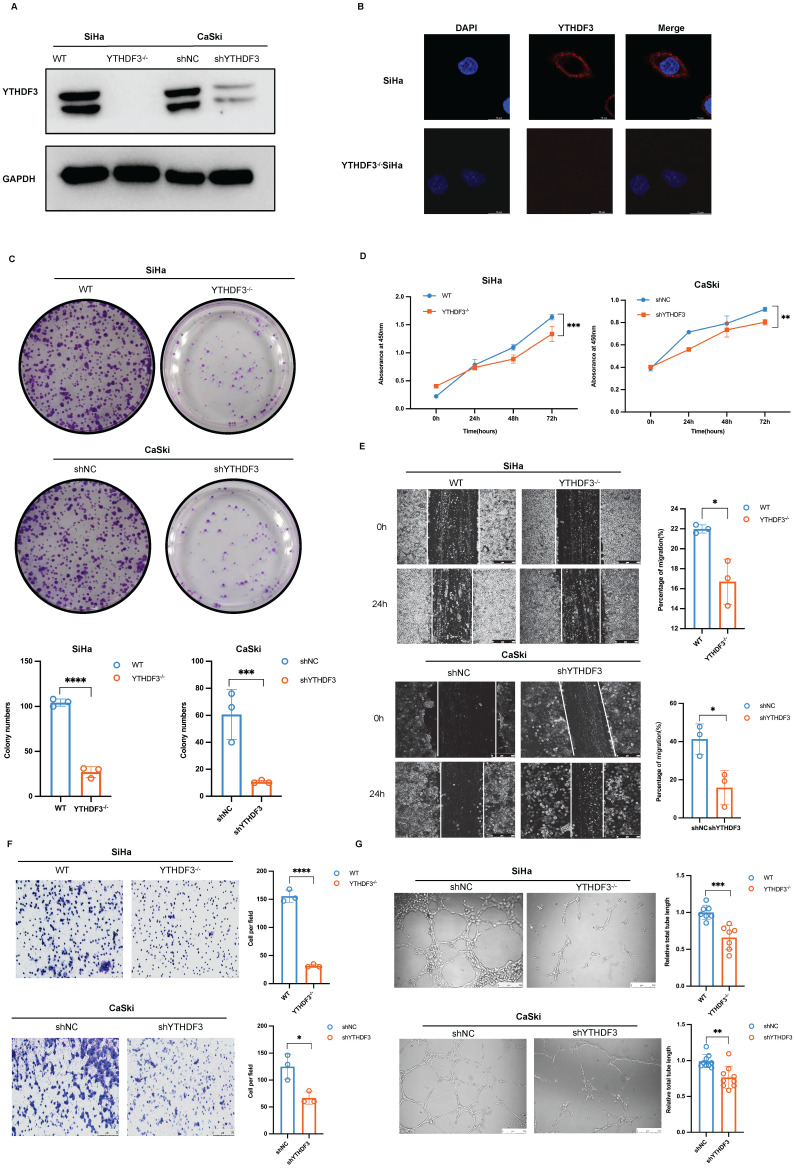
** YTHDF3 promotes CCa proliferation, invasion, and lymphangiogenesis of CCa cells *in vitro*. (A)** The knockdown efficiency of YTHDF3 was validated by western blots. **(B)** The immunofluorescence images exhibited location and expression for YTHDF3 in the SiHa and YTHDF3^-/-^ SiHa cells (under fluorescence microscope at 200 × magnification). **(C)** Colony assays were conducted to investigate the effects of the YTHDF3 on the colony-formation ability of SiHa and CaSki cell lines. **(D)** CCK-8 assays indicated the impacts of YTHDF3 on the proliferation abilities of SiHa and CaSki cell lines. **(E)** Wound healing assays showed the effects of YTHDF3 on the migration abilities of SiHa and CaSki cell lines (under a light field microscope at 100 × magnification). **(F)** Transwell assays performed on the impacts of YTHDF3 on the invasion abilities of SiHa and CaSki cell lines (under light field microscope at 200 × magnification). **(G)** The HLECs were exposed to the culture medium supernatants derived from both the YTHDF3^-/-^ SiHa and the control group. The average length of the tube was quantified (100 × magnification) to evaluate the impact of YTHDF3 on the tube formation of HLECs. The data are shown as the means ± SDs; *P < 0.05; **P < 0.01; *** *P <* 0.001; **** *P <* 0.0001.

To investigate the potential role of YTHDF3 in promoting the proliferation, migration and invasion of CCa cell lines, we utilized CRISPR/Cas9 technology and lentivirus-based short hairpin RNAs (shRNAs) to establish the stable knockdown of YTHDF3 in CCa cell lines (YTHDF3^-/-^ SiHa and shYTHDF3 CaSki). The knockdown efficiency of YTHDF3 in CCa cells was confirmed by the western blot experiment (Fig. 3A). The location of YTHDF3 was detected by immunofluorescence, indicating its predominant cytoplasmic localization in CCa cell (Fig. 3B). *In vitro* colony formation and CCK8 assays demonstrated that depletion of YTHDF3 impaired cellular proliferative and colonic abilities (Fig. 3C-D). Furthermore, wound-healing assays and invasion experiments revealed that inhibition of YTHDF3 suppressed the migration and invasion capabilities of CCa cells (Fig. 3E-F). To further elucidate the role of YTHDF3 in LN metastasis of CCa, we conducted tube formation analysis using human lymphatic endothelial cells (HLECs). Notably, the depletion of YTHDF3 had a significant inhibitory effect on HLEC tube formation as observed through the co-culture supernatant experiments (Fig. 3G). Altogether, these findings provided compelling evidence supporting the crucial involvement of YTHDF3 in promoting proliferation, migration, invasion, and the lymphangiogenesis of CCa cells *in vitro*.

### YTHDF3 regulates tumorigenesis and LN metastasis of CCa *in vivo*

**Figure 4:**
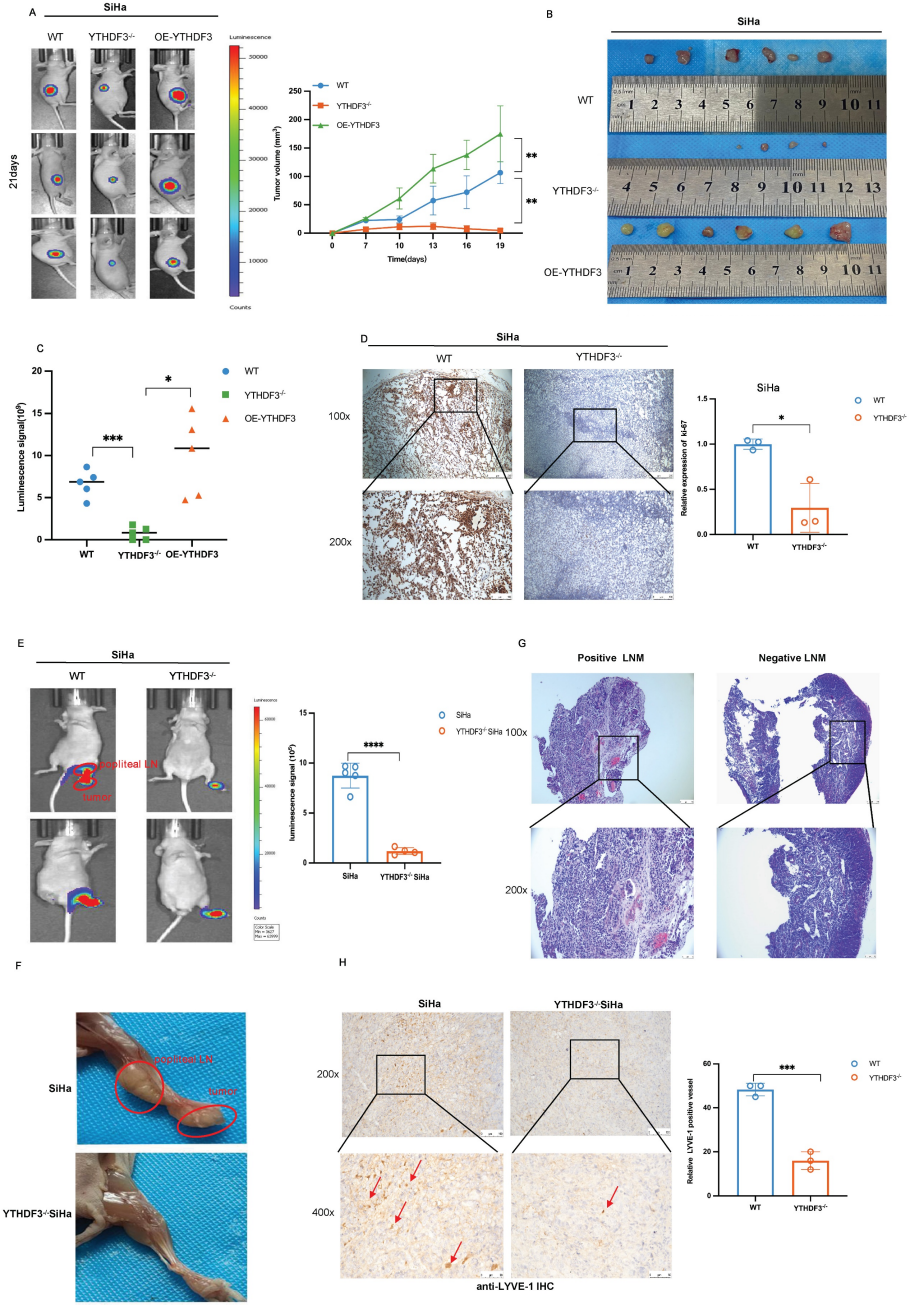
** YTHDF3 regulates tumorigenesis and lymph node metastasis of CCa *in vivo*. (A)** Representative images of bioluminescence for models showed the luciferase signal on the 21^st^ day (n = 6). **(B)** The images showed the volume of dissected tumors in different groups. **(C)** Subcutaneous tumor growth curves of mice in different groups were depicted, the blue line represented the WT-SiHa group, the orange line represented the YTHDF3^-/-^ SiHa, and the green line represented OE-YTHDF3 SiHa group, n = 6 mice. **(D)** Ki-67 was measured in dissected tumors from the two groups by immunohistochemistry, n = 6 mice (under light field microscope at 100 × and 200 × magnifications). **(E)** The bioluminescence image depicted the footpad signal after the injection of knockout YTHDF3 and control SiHa cells. Each dot represented the luciferase signal in the footpad for individual mice on day 35. **(F)** The representative of the image displayed the size of the popliteal lymph node and tumor in the knockout YTHDF3 and control group, n = 5. **(G)** The positive and negative metastatic popliteal lymph nodes were detected by HE staining, n =5 (under light field microscope at 100 × and 200 × magnifications). **(H)** Representative IHC pictures of LYVE-1 were removed from primary tumors. The pictures showed the level of lymph vessels in primary tumors in the YTHDF3^-/-^ SiHa and the control group (under light field microscope at 200 × and 400 × magnifications). The data are shown as the means ± SDs; *P < 0.05; **P < 0.01; *** *P <* 0.001; **** *P <* 0.0001.

To elucidate the functions of YTHDF3 in tumorigenesis, we established subcutaneous xenograft models using YTHDF3-deficient (YTHDF3^-/-^), YTHDF3-overexpression (OE-YTHDF3), and wild-type (WT)-SiHa cells (n = 6/group). Remarkably, the OE-YTHDF3 group exhibited enhanced fluorescent signal in tumors compared to their respective control group, as demonstrated by *In vivo* Imaging System (IVIS) analysis (Fig. 4A). All xenograft models were sacrificed on the 21^st^ day and upon removal of tumors, images showed a significant reduction in the volume of tumor within the YTHDF3^-/-^ groups compared to both the WT- group and OE-YTHDF3 group, indicating that the upregulation of YTHDF3 promoted tumorigenesis *in vivo* (*P* < 0.01) (Fig. 4B-C). The IHC images demonstrated a remarkable downregulation in ki-67 percentage within the YTHDF3^-/-^ groups (Fig. 4D), suggesting that YTHDF3 knockdown inhibited proliferation ability *in vivo*.

To further identify the role of YTHDF3 in LN metastasis of CCa, we established LN metastasis models by injecting YTHDF3^-/-^ SiHa or SiHa cells into the footpads (n = 5/group). The YTHDF3^-/-^ SiHa group exhibited a significant reduction in both luciferase-positive popliteal LNs and the rate of LN metastasis (Fig. 4E-F). Additionally, histological examination using H&E staining confirmed the occurrence of LN metastasis (Fig. 4G).

Importantly, IHC analysis revealed a dramatic decrease in LYVE-1 expression, a specific marker for lymphatic vessels, in the YTHDF3-deficient group, indicating that silencing of YTHDF3 effectively suppressed lymphangiogenesis of CCa (Fig. 4H). Taken together, these findings suggested that YTHDF3 knockdown effectively suppressed lymphangiogenesis and LN metastasis *in vivo*.

### Unveiling m^6^A profiles and identification of YTHDF3-regulated LRP6 expression in CCa cells

**Figure 5:**
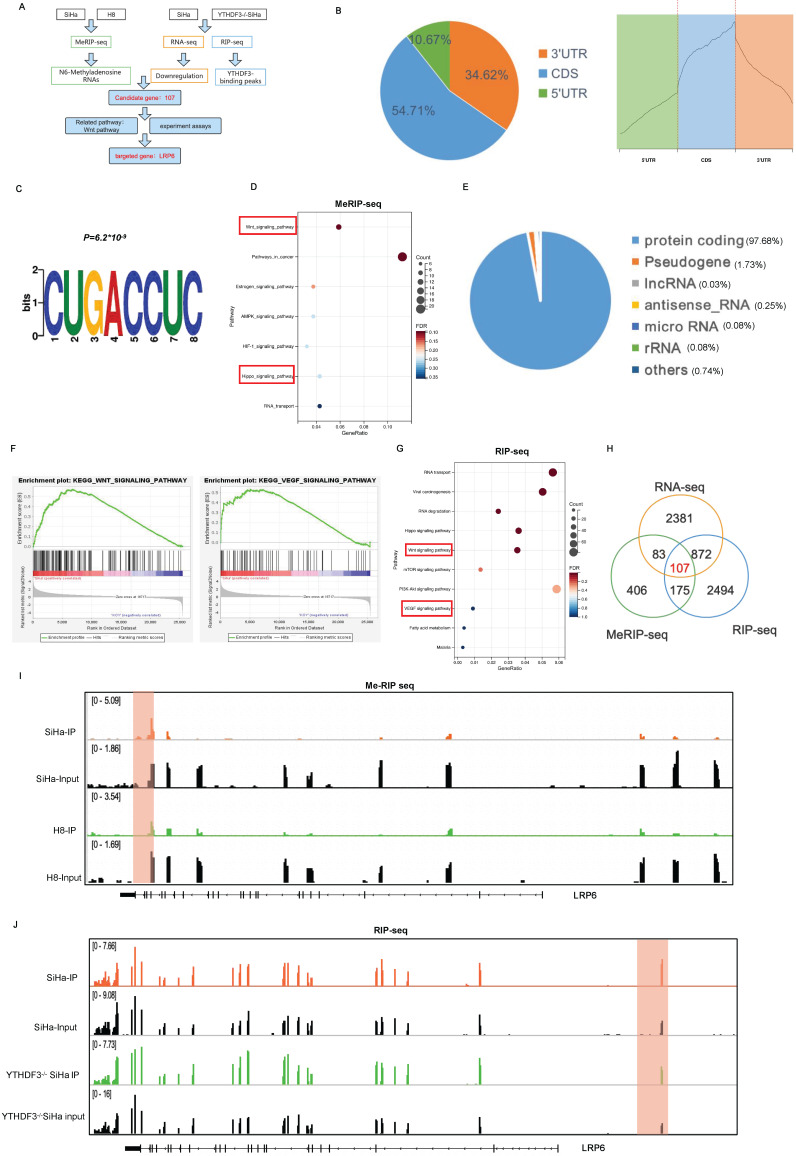
** Identification of YTHDF3-regulates genes by RNA-seq, RIP-seq, and MeRIP-seq. (A)** Flow chart of the screening and validation of the correlation between the expression of YTHDF3 and that of its downstream target LRP6. **(B)** Percentages and metagene profiles of RNA species by m^6^A modifications in 5' untranslated regions, coding sequences, stop codon, and 3' untranslated region. **(C)** The m^6^A motif was detected by MEME motif analysis of the MeRIP-seq. **(D)** The upregulated of m^6^A-associated genes enriched the Wnt signaling pathway and Hippo signaling pathway through KEGG analysis of MeRIP-seq (SiHa and H8 cell lines). **(E)** Distribution of YTHDF3 bound with mRNA was identified by RIP-seq. **(F)** Gene set enrichment analysis plots (GSEA) of RNA-seq revealed that the DEGs were enriched in the Wnt signalling and VEGF signalling pathways. **(G)** KEGG analysis of RIP-seq displayed that the YTHDF3- bound genes were enriched in the Wnt signalling and VEGF signalling pathways. **(H)** Overlapping analysis of genes identified by MeRIP-seq, RIP-seq, and RNA-seq. **(I)** IGV software was utilized to show the peaks with m^6^A enrichment in SiHa and H8 cell lines. The y-axis shows normalized reads distribution for input (black) and m^6^A IP (orange or green). **(J)** IGV image of RIP-seq was used to display the binding region of YTHDF3-LRP6. The y-axis shows normalized reads distribution for input (black) and IP (orange or green).

YTHDF3 functions as an m^6^A reader by binding to and modulating the translation efficiency of m^6^A-methylated transcripts. However, the precise function of the YTHDF3-mediated m^6^A modification in CCa remains to be elucidated. To unveil the direct targets of YTHDF3, we then cross-referenced the transcripts identified by RNA-seq, MeRIP-seq, and RIP-seq (Fig. 5A). MeRIP-seq was initially performed on CCa cells (SiHa) and immortal cervical epithelium cells (H8), revealing that the distribution of m^6^A peaks in CCa cells enriched within coding sequences (CDS) (Fig. 5B). Moreover, MEME algorithm analysis provided further support for the enrichment of the m^6^A modification in mRNAs through the confirmation of the m^6^A consensus motif (CUGACCUC) (Fig. 5C). Subsequently, the KEGG analysis indicated that the upregulated m^6^A-associated genes were enriched in both Wnt and Hippo signaling pathways (Fig. 5D). We next performed RNA-sequencing (RNA-seq) and RNA immunoprecipitation sequencing (RIP-seq) on YTHDF3^-/-^ SiHa and WT-SiHa cells. Through this analysis, we identified a majority of protein-coding genes bound with YTHDF3 as determined by gene-specific RIP-seq assays (Fig. 5E). Enrichment analysis of sequencing data indicated significant alterations in Wnt and VEGF signaling pathways upon depletion of YTHDF3, suggesting a positive correlation between YTHDF3 expression and activation of these pathways (Fig. 5F-G). The visual representation depicted that 282 genes with m^6^A modification were bound to YTHDF3, among which 107 (37.94%) genes exhibited a decline in expression upon loss of YTHDF3 (Fig. 5H). Surprisingly, out of the 107 genes analyzed, the LRP6 had a significant association with the Wnt-signaling pathway and demonstrated substantial alterations during the MeRIP-seq, RNA-seq and RIP-seq analysis (Supplementary Table 4. Importantly, the m^6^A peaks on LRP6 mRNA were observed in both SiHa and H8 cells (Fig. 5I), while RIP-seq analysis on WT-SiHa and YTHDF3^-/-^ SiHa displayed the precise binding site of YTHDF3 on LRP6 mRNA (Fig. 5J). Collectively, these findings suggested that LRP6 may be a crucial target of YTHDF3 in CCa cells.

### YTHDF3 facilitates the expression of LRP6 in an m^6^A-dependent manner

**Figure 6:**
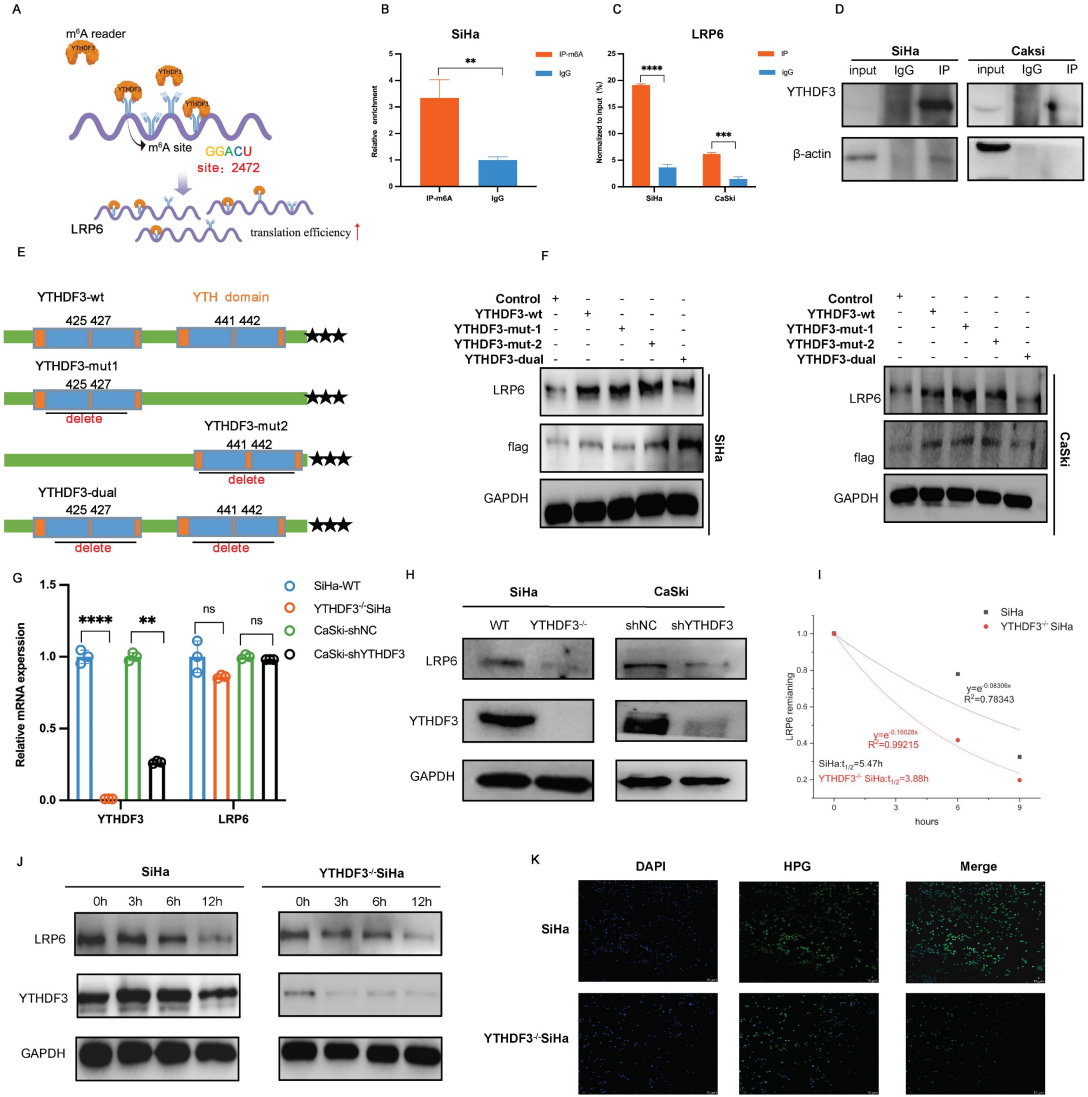
** YTHDF3 promotes *LRP6* mRNA translation in an m^6^A-dependent manner. (A)** The schematic diagram drawn by Figdraw indicated that YTHDF3 is directly bound with LRP6 to promote its translation efficiency. **(B)** MeRIP-qPCR analysis revealed the m^6^A enrichment of LRP6 mRNA by using anti-IgG and anti-m^6^A antibodies in SiHa. **(C-D)** RIP-qPCR analysis showed that the LRP6 mRNA was bound with the YTHDF3 protein. **(E)** Schematic image showing the mutation site (425-427, 441-442) in the wild-type (YTHDF3-wt) and mutant (YTHDF3-mut). **(F)** Western blot analysis was conducted to measure the expression of Flag-YTHDF3 and LRP6 in CCa cells after transfected with control, Flag-YTHDF3-wt or Flag-YTHDF3-mut plasmids for 48 h. **(G)** The relative mRNA level of LRP6 in the knockdown YTHDF3 of CCa cells. **(H)** Western blot analysis showed the protein expression of LRP6 in the knockdown YTHDF3 of CCa cells. **(I)** There was no significant difference in the half-life (t^1/2^) of LRP6 mRNA after treatment with Act D for 0, 3, 6 h and 9 h in between YTHDF3^-/-^ SiHa and Ctrl cells. **(J)** The protein levels of LRP6 were examined after incubation with CHX for 0 h, 3 h, 6 h, and 12 h (100 ug/ml). **(K)** The fluorescence intensity was utilized to evaluate HPG incorporation in the YTHDF3^-/-^ SiHa and the control group. The data are shown as the means ± SDs; *P < 0.05; **P < 0.01; *** *P <* 0.001; **** *P <* 0.0001; ns, nonsignificant.

The schematic image showed that YTHDF3 promoted the translational efficiency of LRP6 by the m^6^A binding site, leading to the elevation of LRP6 protein (Fig. 6A). To ascertain the m^6^A modification level of the LRP6 mRNA in CCa, gene-specific m^6^A-qPCR was performed using SiHa cells. Indeed, discernible m^6^A peaks were detected among LRP6 mRNA (Fig. 6B). Subsequent RIP-qPCR analysis showed significant enrichment of YTHDF3-bound LRP6 mRNA compared to immunoglobulin (IgG) control (Fig. 6C-D). Moreover, we generated the Flag-tagged YTHDF3 mutants (YTHDF3-mut1; YTHDF3-mut2; YTHDF3-dual) harbouring specific amino acid substitutions (425-427;441-442) and transfected them into both the SiHa and CaSki cells. Western blots analysis demonstrated that YTHDF3-mutant groups, especially YTHDF3-dual groups, significantly decreased LRP6 protein expression due to m^6^A reader activity deficiency (Fig. 6E-F). As expected, YTHDF3 knockdown remarkedly diminished the protein levels of LRP6 while leaving its mRNA unaffected (Fig. 6G-H). Further analysis confirmed that YTHDF3 did not impact the LRP6 mRNA stability (Fig. 6I). To preclude any potential impact of YTHDF3 on LRP6 protein stability, SiHa and CaSki cells were treated with cycloheximide (CHX) to block translation. Our findings indicated that the absence of YTHDF3 did not affect the protein stability of LRP6 (Fig. 6J). To further investigate whether YTHDF3 expression influences translation efficiency, we conducted nascent protein synthesis assays. The results indicated a reduction in Homopropargylglycine (HPG) fluorescence intensity upon suppression of YTHDF3 in SiHa cells (Fig. 6K). These findings collectively implied that YTHDF3-mediated m^6^A modification could regulate the translational efficiency of LRP6 rather than its mRNA or protein stability.

### YTHDF3-mediated LRP6 upregulation promotes LN metastasis via reprogramming FA metabolism in CCa

**Figure 7:**
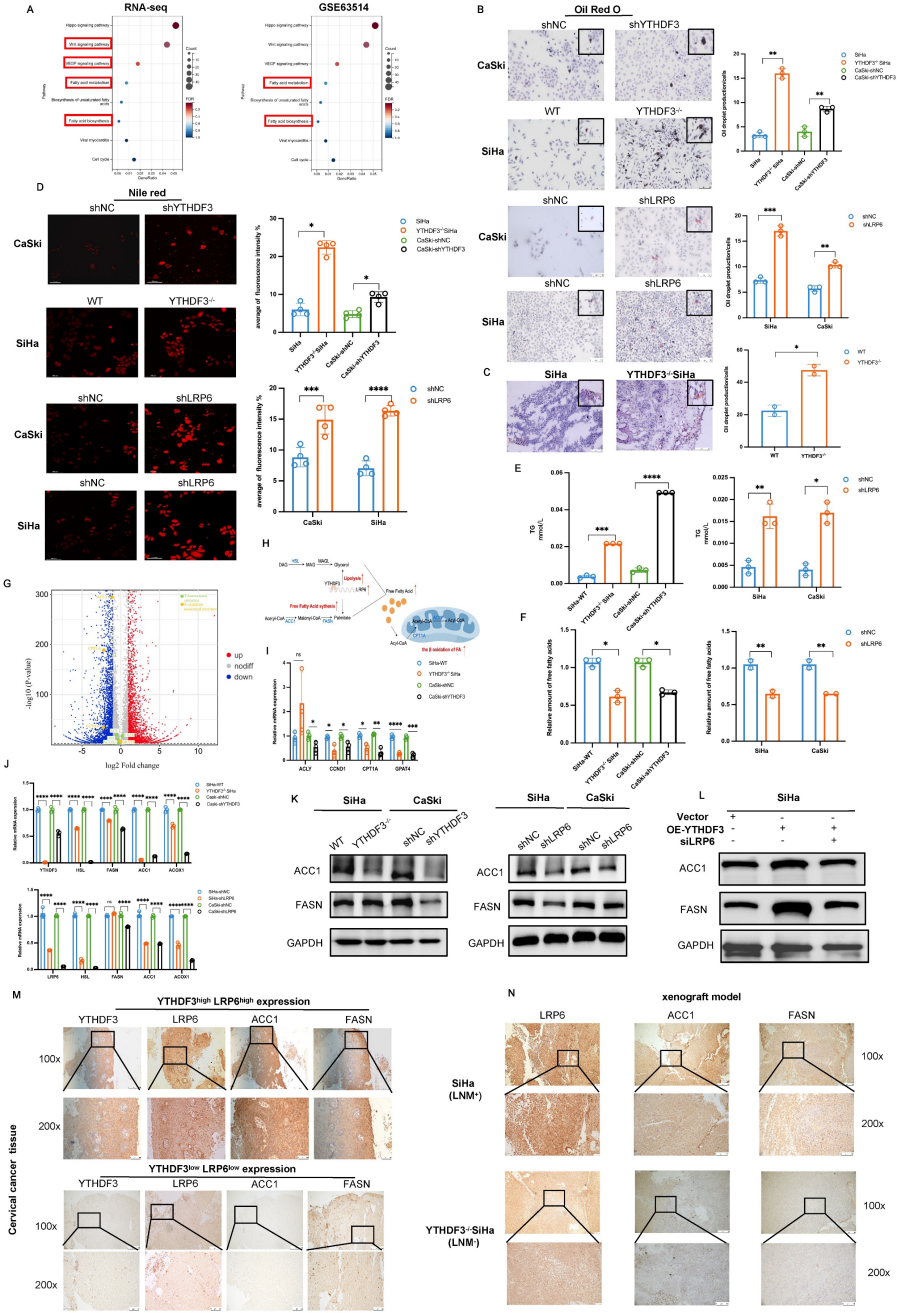
** Effects of YTHDF3 and LRP6 on FA metabolism in CCa. (A)** KEGG of the RNA-seq and the GSE63514 displayed that the DEGs were enriched in Fatty acid metabolism and Fatty acid synthesis.** (B)** LDs (the red) were detected in the inhibition of YTHDF3-mediated LRP6 of the CCa cell line by Oil red staining. **(C)** LDs (the red) were detected in the dissected tumor of LN metastasis models by Oil Red staining. **(D)** LDs (the red) were detected in the suppression of the YTHDF3-mediated LRP6 of the CCa cells through Nile Red staining.** (E)** The production of TG was upregulated by the YTHDF3 knockdown or LRP6-silenced CCa cells.** (F)** The Free Fatty acids were decreased in the YTHDF3-deficient or LRP6-silenced CCa cells.** (G)** The volcano map showed that the ACC1, FASN, HSL, ACOX1 (the fatty acid-associated enzymes), ACLY, CCND1, CPT1A, and GPAT4 (the β-oxidation associated enzymes) were downregulated in the knockout YTHDF3 of SiHa cell compared with the control cell.** (H)** The schematic image explained the relationship between the YTHDF3-LRP6 axis, free fatty acids and β-oxidation. The YTHDF3-LRP6 axis could promote the expression of HSL, ACC1 and FASN (FA-associated enzymes) and the expression of ACLY and CPT1A (β-oxidation associated enzymes). **(I)** The RNA level of ACLY, CCND1, CPT1A, and GPAT4 was downregulated in the inhibition of the YTHDF3-LRP6 axis of SiHa and CaSki cells. **(J)** The RNA expression of key enzymes (ACC1, HSL, FASN, ACOX1) displayed upon the inhibition of the YTHDF3-LRP6 axis in CCa cells. **(K)** The protein levels of key enzymes (ACC1, FASN) displayed upon the inhibition of the YTHDF3-LRP6 axis in CCa cells. **(L)** Western blots showed the protein levels of key enzymes (ACC1, FASN) in the knockdown LRP6 SiHa cells after overexpressing YTHDF3.** (M)** Representative IHC images of the FASN and ACC1 under the YTHDF3^low^LRP6^low^group and YTHDF3^high^LRP6^high^group in the CCa tissues (under light field microscope at 100 × and 200 × magnifications).** (N)** Representative IHC images displayed the protein level of LRP6, ACC1 and FASN in the LN metastasis models (SiHa and YTHDF3^-/-^ SiHa) (under a light field microscope at 100 × magnifications and 200 × magnifications). The data are shown as the means ± SDs; *P < 0.05; **P < 0.01; *** *P <* 0.001; **** *P <* 0.0001; ns, nonsignificant

LRP6, acting as a co-receptor in the Wnt/ß-catenin signalling pathway, plays crucial roles in LDL uptake and lipid homeostasis regulation 20. Recent research has demonstrated that Wnt signalling can modulate lipogenesis and FA metabolism 21. In this study, bioinformatics analysis and experimental investigations were carried out to ascertain whether YTHDF3-mediated LRP6 upregulation contributed to the reprogramming of lipid metabolism in CCa. KEGG analysis of RNA-seq data (WT-SiHa and YTHDF3^-/-^ SiHa) revealed that the DEGs were significantly enriched in pathways related to FA metabolism (Fig. 7A). This finding was further supported by GEO datasets (GSE63514), as shown in Figure 7A. Additionally, the GSEA analysis of RNA-seq also demonstrated that DEGs had an enrichment in FA metabolism Fig.S1E). By quantifying intracellular lipid droplets (LDs) using Oil Red and Nile Red staining, we verified that the inhibition of YTHDF3-mediated LRP6 promoted the production of LDs *in vitro* (Fig.7B, Fig. 7D). In addition, we examined LDs in LN metastasis models and observed an augmented accumulation of LDs *in vivo* due to YTHDF3 deficiency (Fig. 7C). The breakdown of cellular triglycerides (TG) into glycerol and free fatty acids (FA) is indicative of lipolytic capacity (22. Our findings demonstrated that YTHDF3 downregulation significantly increased the production of TG while concurrently downregulating FA in CCa cells (Fig. 7E-F). The above results indicated the crucial role played by YTHDF3-mediated LRP6 in facilitating lipolysis in CCa cells. Accumulating evidence suggested that altered FA metabolism in cancer cells confers certain advantages for LN metastasis 23,24. The key enzymes involved in FA metabolism and β-oxidation were found to be downregulated in the YTHDF3^-/-^ SiHa compared with the control group, as shown in the volcano plot (Fig. 7G). The schematic image illustrated the effect of the YTHDF3-LRP6 axis on lipolysis and FA β-oxidation (Fig. 7H). In the GSE7410 dataset, we observed a remarkable upregulation of ACC1 in CCa with LN metastasis compared to those without LN metastasis Fig. S1F). Pearson correlation analysis was conducted on the TCGA database, revealing positive correlations between key FA enzymes (HSL, FASN, ACC1, ACOX1), key β-oxidation enzyme (ACLY, CCND1, CPT1A, GPAT4), and YTHDF3-LRP6 (Fig. S1G), (Fig. S2A). Furthermore, depletion of YTHDF3 resulted in transcriptional suppression of CCND1, CPT1A and GPAT4 as confirmed by RT-PCR analysis (Fig. 7I). Our findings indicated that inhibition of YTHDF3-mediated LRP6 resulted in the downregulation of ACC1 and FASN expression in CCa cells (Fig.7J-K). Importantly, western blot results showed that silencing LRP6 attenuated the facilitative effect of overexpressed YTHDF3 on increasing ACC1 and FASN protein expression in SiHa (Fig. 7L). Interestingly, we observed a positive correlation between the levels of YTHDF3 and LRP6 in CCa tissues with those of ACC1 and FASN (Fig. 7M). Together, our assessment of FA metabolic enzymes in LN metastasis models revealed that the LNM (+) groups exhibited elevated expression of ACC1 and FASN compared to their LNM (-) counterparts (Fig. 7N). In summary, our findings suggested that YTHDF3-mediated LRP6 could play a pivotal role in facilitating LN metastasis in CCa by reprogramming FA metabolism.

### YTHDF3 activates the LRP6-YAP-VEGF-C axis for LN metastasis in CCa

**Figure 8:**
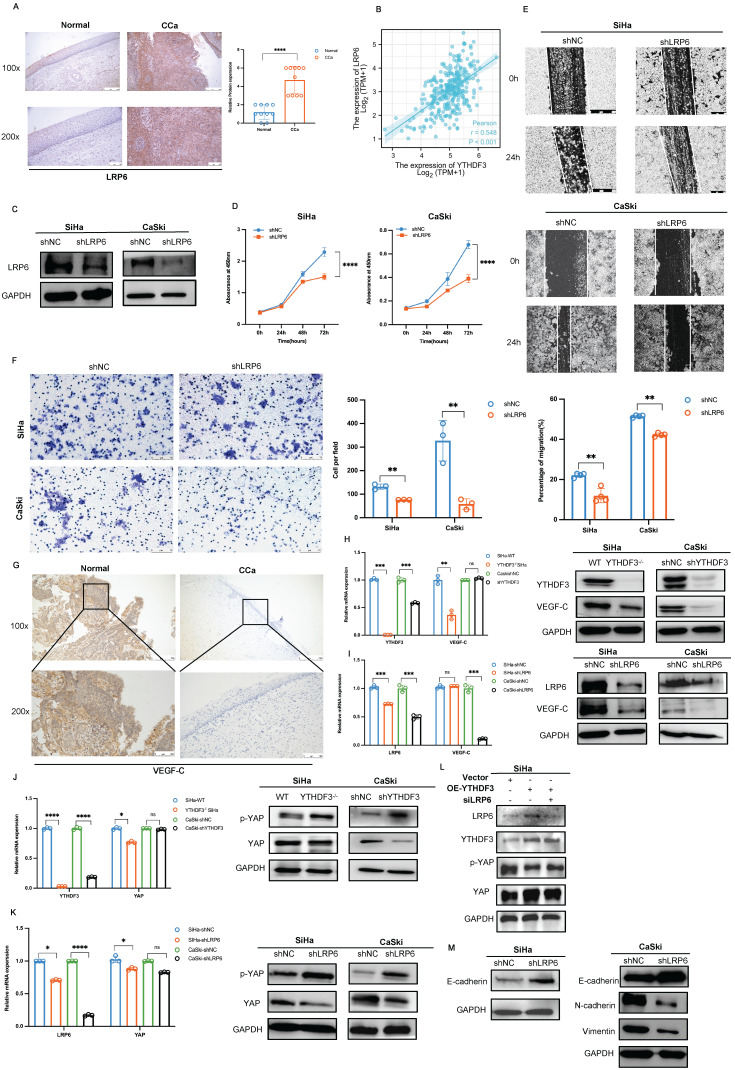
** YTHDF3 accelerates CCa malignant progression and lymph node metastasis by activating Wnt/β-catenin through LRP6s. (A)** Representative IHC images showing the LRP6 protein in primary CCa (n = 10) and normal cervical epithelium (n = 10). **(B)** Pearson correlation revealed that the upregulation of LRP6 was positively parallel with an increase in YTHDF3 expression in CCa by the Xiantao website. **(C)** Western blots confirmed that the protein LRP6 was downregulated after using lentivirus (shLRP6) in CCa cells. **(D)** The inhibition of LRP6 significantly impaired the proliferation of SiHa and CaSki cells by CCK-8 assays. **(E)** The downregulation of LRP6 in SiHa and CaSki cells inhibited the abilities of migration by wound healing experiments (under a light field microscope at 100 × magnifications). **(F)** Transwell invasion assays revealed that the knockdown of LRP6 impaired the abilities of invasion of SiHa and CaSki cells (under light field microscope at 200 × magnifications). **(G)** The representative IHC image showed the expression of VEGF-C in primary CCa (n = 3) and normal cervical epithelium (n = 3) (under a light field microscope at 100 × and 200 × magnifications). **(H)** The qPCR analysis and Western blots displayed the expression of VEGF-C in the YTHDF3-deficient CCa cells. **(I)** The qPCR analysis and Western blots displayed the expression of VEGF-C in the LRP6-deficient CCa cells. **(J)** Western blot and RT-qPCR analysis were performed to measure the protein and RNA levels of p-YAP and YAP in YTHDF3-deficient SiHa and CaSki cells. **(K)** Western blot and RT-qPCR analysis were performed to measure the protein and RNA levels of p-YAP and YAP in the knockdown LRP6 of SiHa and CaSki cells. **(L)** Western blot analysis was evaluated to the protein levels of p-YAP and YAP in the knockdown LRP6 SiHa cells after overexpressing YTHDF3. **(M)** The protein levels of N-cadherin, E-cadherin, and Vimentin were displayed in the LRP6 deficient of CCa cell lines. The data are shown as the means ± SDs; *P < 0.05; **P < 0.01; *** *P <* 0.001; **** *P*<0.0001; ns, nonsignificant.

LRP6 serves as a member of the Wnt/β-catenin signalling pathway, driving cancer progression (1. Nevertheless, its contribution to CCa development with LN metastasis remains elusive. Therefore, we conducted IHC experiments and found that LRP6 expression was remarkably upregulated in CCa tissues (n = 10) compared to the normal cervical squamous epithelium (n = 10) (*P* < 0.0001; Fig. 8A). Furthermore, Pearson correlation analysis revealed a positive correlation between LRP6 and YTHDF3 expression in CCa based on the TCGA database (Fig. 8B). To explore the biological functions of LRP6 in CCa, we established knockdown cell lines using small hairpin RNAs (Fig. 8C). We found that LRP6 deficiency impaired CCa proliferation abilities (Fig. 8D), while wound healing assay demonstrated that the inhibition of LRP6 significantly impeded its migratory ability in CCa cells (Fig. 8E). Moreover, transwell experiments demonstrated suppression of invasive cells upon deletion of LRP6 (Fig. 8F).

Further IHC analysis revealed a significant increase of VEGF-C in CCa tissues compared to normal cervical tissues, as depicted in Figure 8G. Several studies established the association between metastasis and activation of LRP6-mediated Wnt-β-catenin signalling across various cancers 25. Interestingly, we observed a dramatic decrease in VEGF-C expression upon suppression of the YTHDF3/LRP6 axis in CCa cells (Fig. 8H-I). Emerging evidence has identified the crucial role of YAP in regulating lymphatic metastasis by activating VEGF-C expression 26. As above mentioned, LRP6 can be regulated by the Hippo signaling pathway 27, leading us to speculate that the induction of VEGF-C expression in the LN metastasis of CCa may be attributed to the LRP6/YAP axis. Our Pearson correlation analysis revealed a certain connection between the YTHDF3/LRP6 axis and YAP Fig. S2B). Moreover, we confirmed that the downregulation of YTHDF3 resulted in increased levels of phosphorylated YAP but decreased overall expression of YAP protein in the SiHa and CaSki cells (Fig. 8J). Western blot analysis indicated that silencing of LRP6 expression not only induced YAP phosphorylation but also reduced the YAP protein levels in CCa cells (Fig. 8K). Interestingly, overexpression of YTHDF3 in LRP6 knockdown SiHa cells resulted in increased phosphorylated YAP while exhibiting a decline in YAP expression compared to the OE-YTHDF3 SiHa group (Fig. 8L). Additionally, we found that LRP6 deficiency led to a decrease in N-cadherin and Vimentin expression, while both mRNA and protein levels of E-cadherin increased in CCa cells (Fig. 8M, Fig. S2C). These outcomes indicated that YTHDF3 stimulated LN metastasis and EMT by activating the LRP6/YAP/VEGF-C axis.

## Discussion

LN metastasis is the most prevalent mode of distant metastasis in CCa, necessitating a comprehensive understanding for the development of effective treatment strategies. Unfortunately, the precise mechanism underlying LN metastasis in CCa remains elusive. Nevertheless, accumulating evidence has implicated the m^6^A reader YTHDF3 in various biological processes including translational regulation, viral process, stemness maintenance and metastasis (9,20 28-30. Through analyzing clinicopathological parameters and outcomes from both the TCGA database and tissue microarray analysis (TMA) of CCa samples, we unveiled that upregulation of YTHDF3 in CCa tissues was positively associated with LN metastasis status as well as disease stages. The identification of YTHDF3's function in promoting lymphangiogenesis opens up novel avenues for the development of therapies aimed at curtailing LN metastasis in CCa. In our investigation, we found that aberrant elevation of YTHDF3 significantly enhanced tumorigenicity and LN metastasis in CCa.

Furthermore, by employing RIP-seq and RIP-qPCR techniques, we successfully identified LRP6 as a YTHDF3-bound target gene. Our findings suggested that YTHDF3 enhanced LRP6 translation efficiency through m^6^A modification, as evidenced by an increase in LRP6 protein despite unaltered mRNA levels. LRP6, a member of the low-density lipoprotein receptor family, has been widely recognized for its pivotal role in driving cancer progression via aberrant upregulation 31,32. Consistent with previous studies highlighting the inhibitory effects of Wnt signalling suppression on breast cancer metastasis and prostate cancer cell proliferation 33,34. Our study further unveils the oncogenic potential of LRP6 in accelerating the malignant phenotypes and EMT progression of CCa.

Metabolic reprogramming is essential for cancer cells to metastasize, as abnormal lipid intake or production and overactive de novo synthesis can expedite tumor progression 35,36,37. Multiple studies supported the concept that FASN served as a pivotal regulator of lipid metabolism and was closely linked to LN metastasis with CCa 38,39. LRP6 acted as a regulator involved in maintaining lipid homeostasis via nutrient-sensing mTOR pathways while promoting aberrant lipid lipogenesis and regulating fatty acid synthesis in cancer cells 40. The fatty acids are transported to the mitochondria and undergo β-oxidation, resulting in the production of acetyl-CoA. Furthermore, it has been proved that the β-oxidation process of fatty acid plays an important role in lymphangiogenesis 41. Our research not only confirmed that the YTHDF3-m^6^A-LRP6 led to the lipolysis and FA synthesis, but also affected the β-oxidation process. All of the results suggested a potential mechanistic link between FA metabolism and the occurrence of LN metastasis in CCa.

Growing evidence indicated that YAP plays a crucial role in tumor metastasis to lymph nodes through metabolic adaption 42,43. Moreover, activation of the Hippo-YAP/TAZ pathway emerged as a key signal for LN metastasis and lymphangiogenesis 44,45. VEGF-C, a specific growth factor for lymphatic vessels enhances LN metastasis and promotes lymphangiogenesis. Our research revealed that the YTHDF3 could elevate VEGF-C expression in CCa cells. Further investigation into the mechanism underlying the interaction between YTHDF3 and VEGF-C uncovered that LRP6, mediated by YTHDF3, was capable of enhancing YAP transcription by suppressing its phosphorylation. This ultimately leads to upregulated expression of VEGF-C and subsequent LN metastasis in CCa.

## Conclusion

Our research findings indicated that the oncogene YTHDF3 significantly promoted LN metastasis of CCa by modulating fatty acid metabolism. YTHDF3 enhanced LRP6 mRNA translation, leading to increasing LRP6 expression and activating the LRP6-YAP-VEGF-C axis, which subsequently induced lymphangiogenesis and LN metastasis of CCa. Moreover, YTHDF3-mediated LRP6 stimulated lipolysis of lipid droplets and FA synthesis by activating key enzymes FASN and ACC1 involved in FA metabolism. Hence, we inferred that targeting the YTHDF3/m^6^A/LRP6 can reprogram fatty acid metabolism to suppress the LN metastasis in CCa.

## Supplementary Material

Supplementary figures and tables 1-2.

Supplementary table 3.

Supplementary table 4.

## Figures and Tables

**Table 1:** Association between YTHDF3 expression and clinicopathological characteristics of CCa patients

Characteristics	Total	YTHDF3 expression High Low		*P*-value
**Age**				= 0.2061
< 50	105	70	35	
> 50	39	21	18	
**Pathological type**				= 0.0063
Normal	32	27	5	
CINIII	17	7	10	
Carcinoma	95	57	38	
**Clinical stage TNM**				= 0.6573
I	48	27	21	
II	25	15	10	
III	19	13	6	
**Lymph node metastasis**				= 0.0298
Yes	75	45	30	
No	20	6	14	
**m^6^A expression**				< 0.0001
Low expression	60	50	10	
High expression	73	36	37	

The* P* Value was measured by the Chi-square test. *P < 0.05, **P < 0.01, ***P < 0.001, ****P < 0.0001.

**Table 2:** Correlation between m^6^A protein expression and clinicopathological characteristics of CCa patients

Characteristics	Total	m^6^A expression High Low	*P*-value
**Age**			= 0.023
< 50	99	50 49	
> 50	39	11 28	
**Pathological type**			= 0.058
Normal	30	11 19	
CINIII	17	4 13	
Carcinoma	91	47 44	
**Clinical stage TNM**			= 0.527
I	45	22 23	
II	25	12 13	
III	19	12 7	
**Lymph node metastasis**			= 0.250
No	72	35 37	
Yes	19	12 7	

The *P* Value was measured by the Chi-square test. *P < 0.05, **P < 0.01, ***P < 0.001, ****P < 0.0001.
