# The inhibition of YTHDF3/m^6^A/LRP6 reprograms fatty acid metabolism and suppresses lymph node metastasis in cervical cancer: Erratum

**DOI:** 10.7150/ijbs.115928

**Published:** 2025-06-05

**Authors:** Sheng Zhong, Quanwei Guo, Xiaona Chen, Xiaomin Luo, Yufei Long, Tuotuo Chong, Ming Ye, Hui He, Anwei Lu, Keyi Ao, Minuo Yin, Aimin Xu, Xin Li, Yi Hao, Xia Guo

**Affiliations:** 1 Shenzhen Key Laboratory of Viral Oncology; Department of Science and Innovation, Shenzhen Hospital, Southern Medical University, Shenzhen, China.; 2 The Third School of Clinical Medicine, Southern Medical University Guangzhou, China.; 3 Department of Thoracic Surgery, Shenzhen Hospital, Southern Medical University, Shenzhen, China.; 4 Department of Pathology, Affiliated Tumor Hospital of Xinjiang Medical University, Urumqi, China.; 5 Department of Pathology, Shenzhen Hospital, The University of Hong Kong, Shenzhen, China.; 6 Department of Obstetrics and Gynecology, Shenzhen Hospital of Southern Medical University, Shenzhen, China.; 7 Department of Medicine, University of Hongkong, Hongkong, China.; 8 Department of Ultrasound, South China Hospital of Shenzhen University, Shenzhen, China.

In our paper, the author discovered an error in Figure 8F. We identified that the Figure 8F involved the misused image. Following a thorough and careful re-examination of the original raw data, we have replaced these mislabeled images from the other correct images. The original data has been checked and analyzed again to ensure that the conclusions remain reliable and unaffected by this error. All contributing authors have unanimously approved this correction. We sincerely apologize for any inconvenience caused by this oversight in our work. We remain committed to upholding the highest standards of research integrity in our work.

Figure 8F should be corrected as follows.

## Figures and Tables

**Figure 8:**
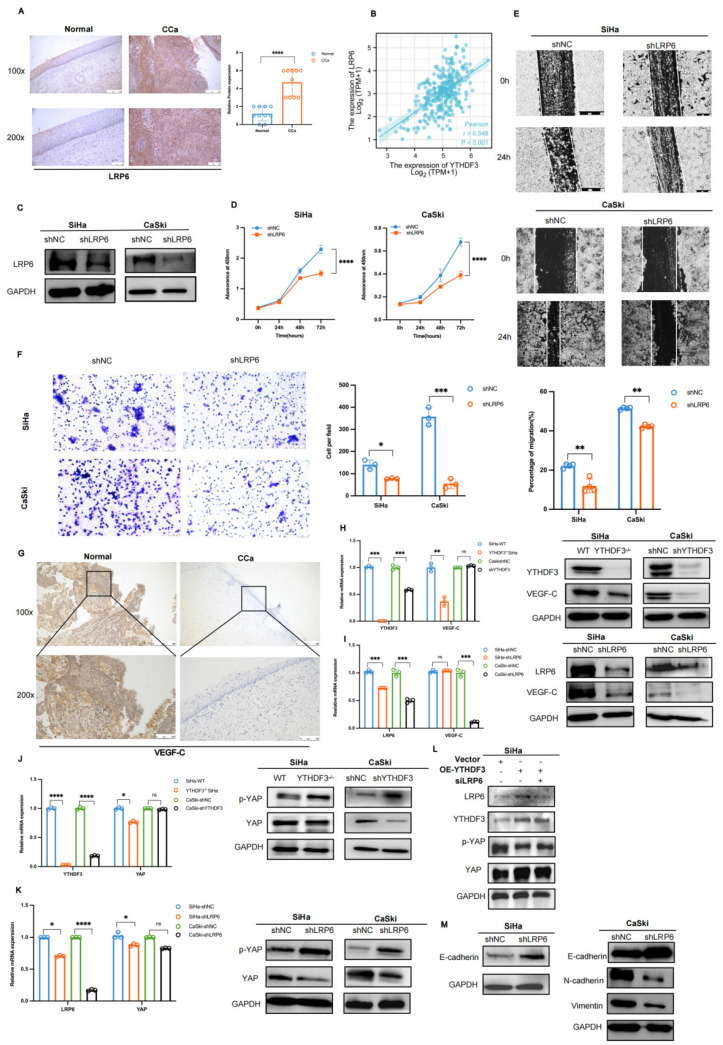
Correct image.

