# Supplementary material for: The inhibition of YTHDF3/m6A/LRP6 reprograms fatty acid metabolism and suppresses lymph node metastasis in cervical cancer
Source: Int J Biol Sci. 2024 Jan 12;20(3):916–36. doi: 10.7150/ijbs.87203 (PMC10797697; doi:10.7150/ijbs.87203)
Supplement: Supplementary file 1 — Supplementary figures and tables 1-2. [file ijbsv20p0916s1.pdf]

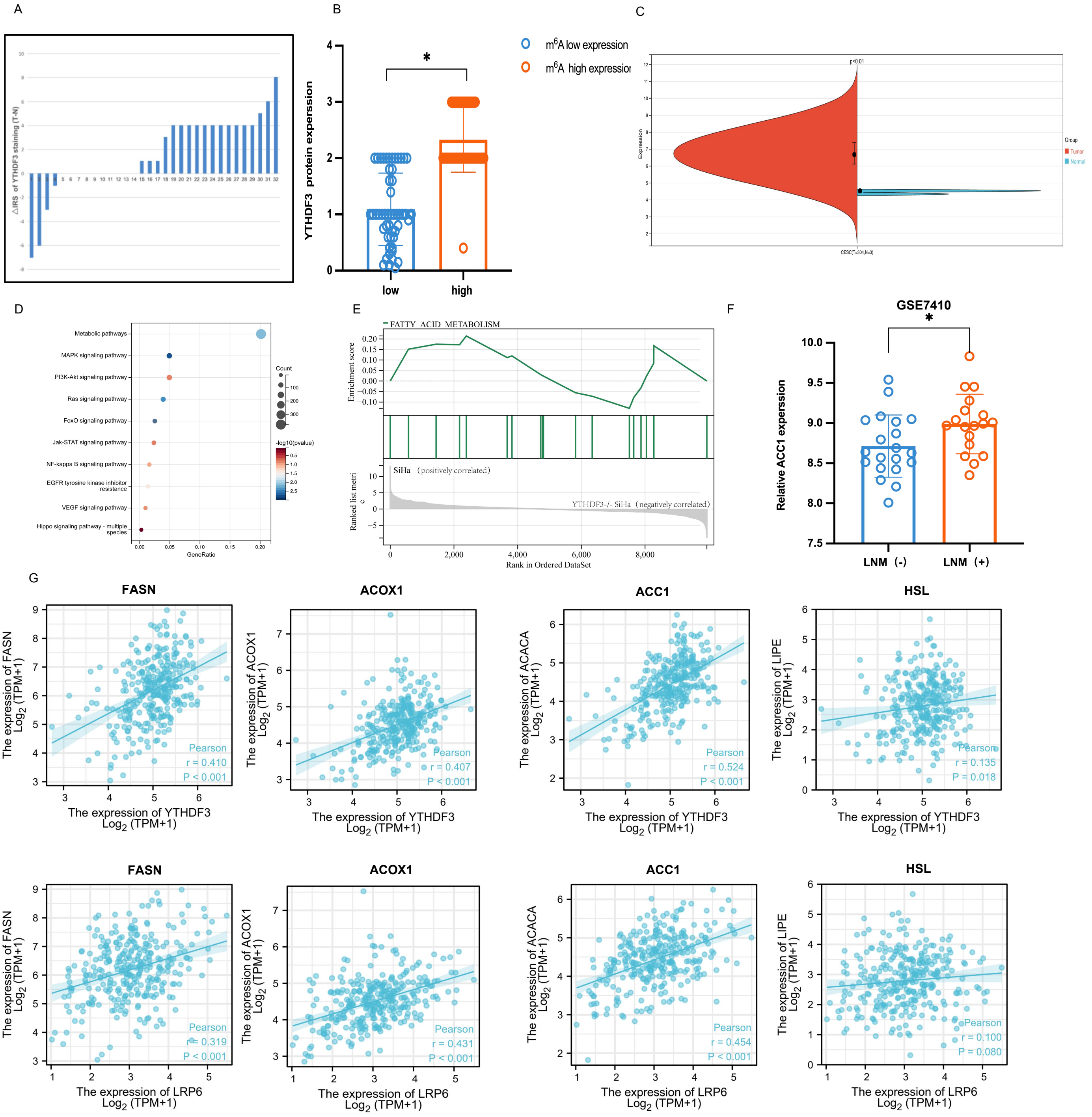

A
△IRS of YTHDF3 staining (T-N)
B
YTHDF3 protein experssion
low
high
*
m6A low expression
m6A high expression
C
p<0.01
Expression
Group
Tumor
Normal
CESC(T=304,N=3)
D
Metabolic pathways
MAPK signaling pathway
PI3K-Akt signaling pathway
Ras signaling pathway
FoxO signaling pathway
Jak-STAT signaling pathway
NF-kappa B signaling pathway
EGFR tyrosine kinase inhibitor resistance
VEGF signaling pathway
Hippo signaling pathway - multiple species
Count
-log10(pvalue)
GeneRatio
E
FATTY_ACID_METABOLISM
Enrichment score
Ranked list metric
SiHa (positively correlated)
YTHDF3-/- SiHa (negatively correlated)
Rank in Ordered DataSet
F
GSE7410
Relative ACC1 experssion
LNM (-)
LNM (+)
G
FASN
ACOX1
ACC1
HSL
The expression of FASN Log2 (TPM+1)
The expression of ACOX1 Log2 (TPM+1)
The expression of ACACA Log2 (TPM+1)
The expression of LIPE Log2 (TPM+1)
The expression of YTHDF3 Log2 (TPM+1)
Pearson
r = 0.410
P < 0.001
r = 0.407
P < 0.001
r = 0.524
P < 0.001
r = 0.135
P = 0.018
The expression of LRP6 Log2 (TPM+1)
r = 0.319
P < 0.001
r = 0.431
P < 0.001
r = 0.454
P < 0.001
r = 0.100
P = 0.080

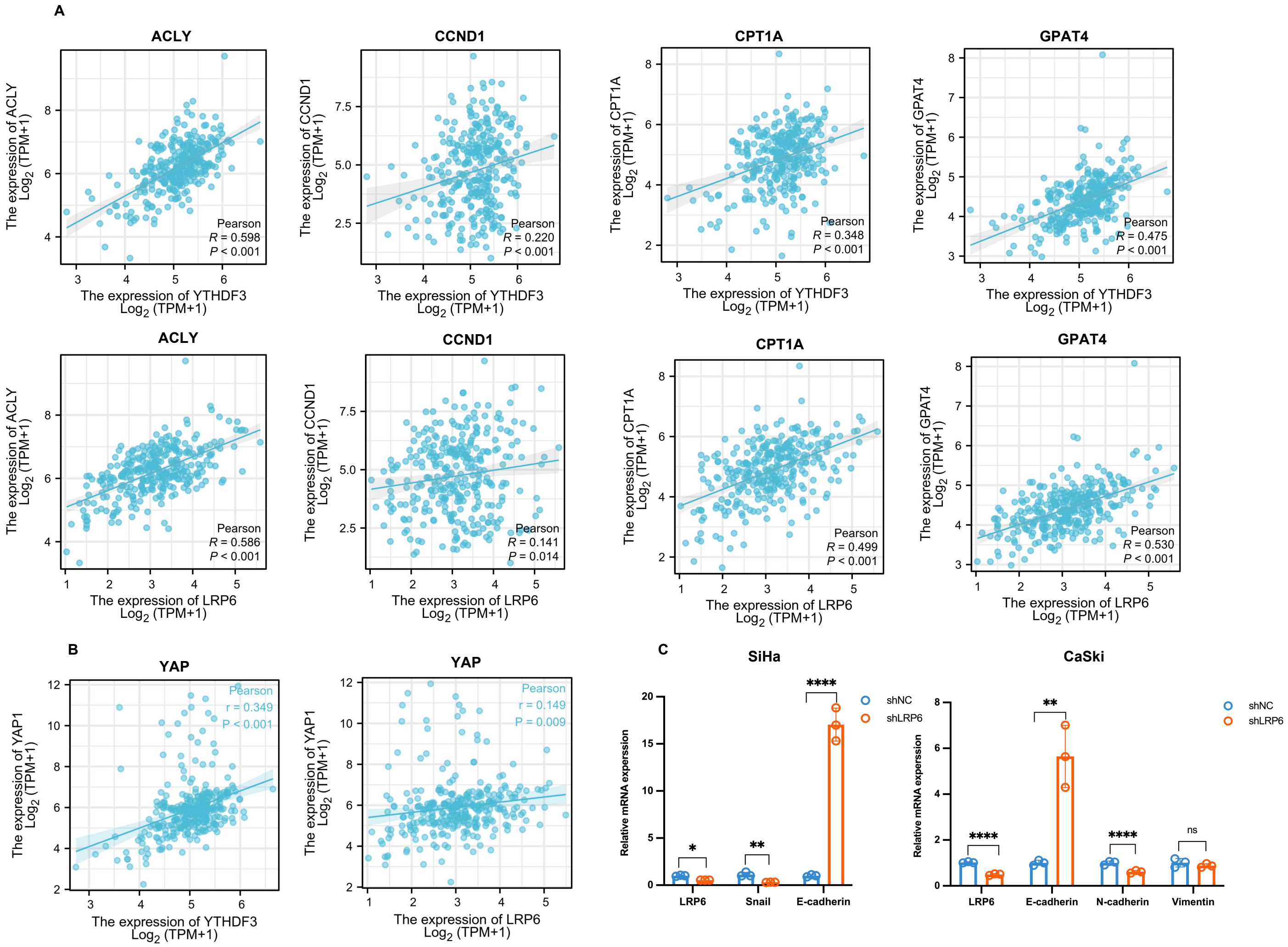
A
ACLY
CCND1
CPT1A
GPAT4
The expression of ACLY Log2 (TPM+1)
The expression of CCND1 Log2 (TPM+1)
The expression of CPT1A Log2 (TPM+1)
The expression of GPAT4 Log2 (TPM+1)
The expression of YTHDF3 Log2 (TPM+1)
The expression of LRP6 Log2 (TPM+1)
Pearson R = 0.598 P < 0.001
Pearson R = 0.220 P < 0.001
Pearson R = 0.348 P < 0.001
Pearson R = 0.475 P < 0.001
Pearson R = 0.586 P < 0.001
Pearson R = 0.141 P = 0.014
Pearson R = 0.499 P < 0.001
Pearson R = 0.530 P < 0.001
B
YAP
The expression of YAP1 Log2 (TPM+1)
Pearson r = 0.349 P < 0.001
Pearson r = 0.149 P = 0.009
C
SiHa
CaSki
Relative mRNA expression
Relative mRNA expersion
shNC
shLRP6
LRP6
Snail
E-cadherin
N-cadherin
Vimentin
*
**
****
ns

Table S1. Antibodies used in the experiments.

Antibody	Source	No. of Catalogue	Dilution		
			WB	IHC	IF
N^6-Methyladenoe	Abcam, USA	220161	1:1000	1:100	
YTHDF3	abcam, USA	190886	1:1000	1:500	1:200
LRP6	Cell Signaling Technology, USA	2560T	1:1000		
LRP6	ABcolonal,China	A13324		1:800	
E-cadherin	Cell Signaling Technology, USA	3195	1:1000		
Vimentin	Protein tech，China	5741	1:1000		
N-cadherin	Protein tech，China	22018-1-AP	1:1000		
p-YAP	Cell Signaling Technology, USA	13008T	1:1000		
YAP	Protein tech，China	135841-1-AP	1:1000	1:400	
PROX1	ABcolonal,China	A9047		1:400	
VEGF-C	ABcolonal,China	A12530		1:500	
GAPDH	YESEN, China	30201ES60	1:5000		
β-actin	YESEN, China	30101ES60	1:5000		
ki-67	Bioss，China	bsm-51754M		1:800	
ACC1	Protein tech，China	21923	1:1000		
FASN	Protein tech，China	10624	1:1000		

Abbreviations: WB: Western blot; IHC: Immunohistochemistry; IF: Immunofluorescence

Table S2. Primers used in the experiments

Gene name		Primer sequence	Application
YTHDF3	F	5'-GGTGCCTGGCATTAGCAGTA-3′	qRT-PCR
	R	5'-CTGGGGGCACACTATTGGTT-3′	
LRP6	F	5'-CCTTGACGTGCCCCTTTCTT-3′	qRT-PCR
	R	5'-CCAATCGCAAGTCCCGTCT-3′	
GAPDH	F	5'-AGAAGGCTGGGGCTCATTTG-3′	qRT-PCR
	R	5'-AGGGGCCATCCACAGTCTTC-3′	
E-Cadherin	F	5'-ATGCAGAAACTGGCATCCTCA-3′	qRT-PCR
	R	5'-AGTCCTCGGACACTTCCACT-3′	
N-Cadherin	F	5'-GTTCATACCCTTGTCCCCTGG-3′	qRT-PCR
	R	5'-CCTGGGCAGTGTAGGATGTG-3′	
Vimentin	F	5'-AAACTTAGGGGCGCTCTTGT-3′	qRT-PCR
	R	5'-GAGGGCTCCTAGCGGTTTAG-3′	
Snail	F	5'-TAGCGAGTGGTTCTTCTGCG-3′	qRT-PCR
	R	5'-AGGGCTGCTGGAAGGTAAAC-3′	
VEGF-C	F	5'-GAGGAGCAGTTACGGTCTGTG-3′	qRT-PCR
	R	5'-TCCTTTCCTTAGCTGACACTTGT-3′	
YAP	F	5'-TAGCCCTGCGTAGCCAGTTA-3′	qRT-PCR
	R	5'-TCATGCTTAGTCCACTGTCTGT-3′	
ACC1	F	5'-ATGTCTGGCTTGCACCTAGTA-3′	qRT-PCR
	R	5'-CCCCAAAGCGAGTAACAAATTCT-3′	
ACOX1	F	5-GGCGCATACATGAAGGAGACCT-3′	qRT-PCR
	R	5-AGGTGAAAGCCTTCAGTCCAGC-3′	
FASN	F	5'-AAGGACCTGTCTAGGTTTGATGC-3′	qRT-PCR
	R	5'-TGGCTTCATAGGTGACTTCCA-3′	
HSL	F	5'-TCAGTGTCTAGGTCAGACTGG-3′	qRT-PCR
	R	5'-AGGCTTCTGTTGGGTATTGGA-3′	
ACLY	F	5'-GGACTTCGGCAGAGGTAGAG-3′	qRT-PCR
	R	5'-TGATCAGCTGGTCTGGCTTG-3′	
CPT1A	F	5'-ATGACGGCTATGGTGTGTCG-3′	qRT-PCR
	R	5'-TTCCAGCCCAGCACATGAAC-3′	
CCDN1	F	5'-GATGCCAACCTCCTCAACGA-3′	qRT-PCR
	R	5'--GGAAGCGGTCCAGGTAGTTC3′	
GPAT4	F	5'-CTCCCCCAAGTCCCAGCTTTG-3′	qRT-PCR
	R	5'-CATACTGCGAGTGCTGAGTG-3′	
si-SREBF1#2	anti-sense	CGGAGAAGCTGCCTATCAA	si-RNA
si-SREBF1#3	anti-sense	CAGCCCTGGTCTACCATAA	si-RNA
sh-LRP6	shRNA	CCTGCCCACTACTCTCTTAAT	sh-RNA
sh-YTHDF3	shRNA	CGGCATATTCGCTTAGAAA	sh-RNA
YTHDF3	sgRNA-1	tggtgtatttagtcaacctg	sgRNA
YTHDF3	sgRNA-2	tgggtagctcctcgtaacag	sgRNA
LRP6	F	5'-TTCCTGGTCAGTGATGCCTT-3′	MeRIP-qPCR
	R	5'-CTCCTCTGACTGCCTCCAAC-3′	

LRP6	F	5’-ACACAGACCAAGAGGCAATTT-3′	RIP-qPCR
	R	5’-AACCGTTTCCCCCAATCACA-3′	

Abbreviations: F: Forward; R: Reverse; qRT-PCR: quantitative real-time PCR; RIP: RNA binding protein immunoprecipitation; MeRIP: Methylated RNA immunoprecipitation; si-RNA: Small interfering-RNA; sh-RNA: Short hairpin-RNA
